# Enhancing and Complementary Mechanisms of Synergistic Action of Acori Tatarinowii Rhizoma and Codonopsis Radix for Alzheimer's Disease Based on Systems Pharmacology

**DOI:** 10.1155/2020/6317230

**Published:** 2020-06-25

**Authors:** Shengwei Liu, Cui He, Yuan Liao, Hailin Liu, Wanli Mao, Zhengze Shen

**Affiliations:** ^1^Department of Pharmacy, Yongchuan Hospital of Chongqing Medical University, Chongqing 402160, China; ^2^Chongqing Key Laboratory of Biochemistry and Molecular Pharmacology, School of Pharmacy, Chongqing Medical University, Chongqing 400016, China; ^3^Zhejiang Key Laboratory of Organ Development and Regeneration, College of Life and Environmental Sciences, Hangzhou Normal University, Hangzhou, Zhejiang 310018, China; ^4^Department of Pharmacy, First People's Hospital of Chongqing Liangjiang New District, Chongqing 401121, China; ^5^The People's Hospital of Yongchuan District, Chongqing 402160, China

## Abstract

**Materials and Methods:**

In this study, a systems pharmacology-based strategy was used to elucidate the synergistic mechanism of Acori Tatarinowii Rhizoma and Codonopsis Radix for the treatment of AD. This novel systems pharmacology model consisted of component information, pharmacokinetic analysis, and pharmacological data. Additionally, the related pathways were compressed using the Kyoto Encyclopedia of Genes and Genomes (KEGG) database, and the organ distributions were determined in the BioGPS bank.

**Results:**

Sixty-eight active ingredients with suitable pharmacokinetic profiles and biological activities were selected through ADME screening *in silico*. Based on 62 AD-related targets, such as APP, CHRM1, and PTGS1, systematic analysis showed that these two herbs were mainly involved in the PI3K-Akt signaling pathway, MAPK signaling pathway, neuroactive ligand-receptor interaction, and fluid shear stress and atherosclerosis, indicating that they had a synergistic effect on AD. However, ATR acted on the KDR gene, while CR acted on IGF1R, MET, IL1B, and CHUK, showing that they also had complementary effects on AD. The ingredient contribution score involved 29 ingredients contributing 90.14% of the total contribution score of this formula for AD treatment, which emphasized that the effective therapeutic effects of these herbs for AD were derived from both ATR and CR, not a single herb. Organ distribution showed that the targets of the active ingredients were mainly located in the whole blood, the brain, and the muscle, which are associated with AD.

**Conclusions:**

In sum, our findings suggest that the systems pharmacology methods successfully revealed the synergistic and complementary mechanisms of ATR and CR for the treatment of AD.

## 1. Introduction

More than 25 million people suffer from dementia worldwide, and Alzheimer's disease (AD) accounts for approximately 70% of patients with dementia [1–3]. AD is a progressive neurodegenerative disorder caused by extensive synapse loss, the formation of intracellular neurofibrillary tangles, extracellular deposits of *β*-amyloid peptides (A*β*), inflammation, and oxidative stress in neurons [4–6]. Clinical data also showed that cerebral vascular diseases, especially atherosclerosis, are established risk factors for dementia, particularly AD [7, 8]. All this evidence suggests that the clinical features of AD are diverse and that the pathological factors are complicated. Therefore, current pharmacology focusing on acetylcholine esterase inhibitors and an *N*-methyl-D-aspartic acid receptor (NMDAR) modulator is unsatisfactory for ameliorating the symptoms of AD [9]. For example, donepezil, the most effective drug for the treatment of AD, can only temporarily relieve the symptoms and cannot inhibit the progression of AD [10]. Development of LY450139, which has been investigated in a phase III trial of anti-A*β* drug candidates, has been halted due to its inhibition of the NOTCH protein, even though it can significantly reduce A*β* levels in patients [5]. Currently approved drugs for the treatment of AD, such as rivastigmine and memantine, result in various side effects, such as bradycardia or hepatotoxicity [11–13]. Hence, it is critical to explore more systemic, effective, and harmless anti-AD agents.

Traditional Chinese medicine (TCM) has a long history in the treatment of dementia [3, 14]. For example, Kai-Xin-San, the representative prescription written by Sun Simiao in *Beiji Qianjin Yaofang* during the Tang Dynasty, is a TCM formula with antidementia effects [15]. Modern pharmacological research also verified that active ingredients extracted from Chinese herbs, such as apigenin, *β*-asarone, and huperzine A, exert curative effects against AD in vivo and in vitro; indeed, some of them have been used in clinical trials [15]. Notably, Acori Tatarinowii Rhizoma (ATR, the rhizome of *A. calamus*, also named *Acorus calamus* var*. angustatus*, Shi Chang Pu in Chinese) is widely used to treat memory and cognitive dysfunction due to its anti-inflammatory, antifibrillar amyloid plaque, and anti-tau phosphorylation activities [16–19]. In TCM, Codonopsis Radix (CR, the roots of *C. pilosula*, also named *Codonopsis pilosula* (Franch.) Nannf., Dang Shen in Chinese) functions by reinforcing qi (vital energy), improving the immune system, and reducing blood pressure and also has an antidiabetic effect by reducing the blood glucose level and inhibiting reactive oxygen species (ROS)-induced damage [20, 21]. Injection of CR has neuroprotective effects based on blockade of brain Ca^2+^ accumulation and improvement in superoxide dismutase activity [22]. For enhanced efficiency, CR is usually combined with ATR, such as Tiaoxin Recipe [23] and Shen-Zhi-Ling oral liquid [24], and it is known as a monarch, minister, assistant, and guide in TCM. However, considering the complexity of traditional experimental methods, it is difficult to explore the detailed synergistic mechanism of ATR and CR.

A single formula of TCM usually comprises many herbs and exerts its curative effect by additive and complementary effects of multiple herbs [25]. Therefore, treating AD with TCM shows the following advantages: (1) TCM includes multiple active ingredients that may activate various kinds of biological targets and pathways to provide synergistic and/or additive effects to benefit patients with AD; (2) TCM is derived from natural products so it may have low toxicity and few side effects [26]. However, owing to the multiple and complex characteristics of these formulas, their exact mechanisms of action are still unclear and difficult to determine by traditional experimental assays. For example, the famous formula Shi-Quan-Da-Bu-Tang in TCM improves the intestinal immune system, but its ten single herbs show no such activity, indicating certain synergistic effects among multiple herbs [25]. This result again demonstrated that it is difficult to explore the mechanisms of action of TCM. In recent years, based on chemical, pharmacokinetic, and pharmacological data, the methodology of systems pharmacology has helped us to interpret the details of the synergistic mechanisms in Chinese formulas [27–29]. Systems pharmacology integrates pharmacokinetics synthesis data, target screening, pathway interaction, and network analysis to elucidate the therapeutic mechanisms of drugs from the molecular and cellular levels to the tissue and organism levels [26, 30]. Following a systems pharmacology-based strategy, many reports explain the polypharmacological and synergistic mechanisms and predict the principal ingredients and signaling pathways of herb pairs for various diseases [31–34]. Using this model, Lu et al. revealed that Huangqi and Huanglian exert synergistic therapeutic effects on diabetes mellitus based on five pivotal ingredients [35]. Zhi-Zhu-Wan has efficacious therapeutic effects on functional dyspepsia due to 29 major active components [16]. Based on systems pharmacology, the ingredients from the herb *Cistanche tubulosa* show synergistic effects on neuroinflammation [36], and the compounds rutin and amentoflavone exert synergistic effects on relieving depression [37].

**Figure 1:**
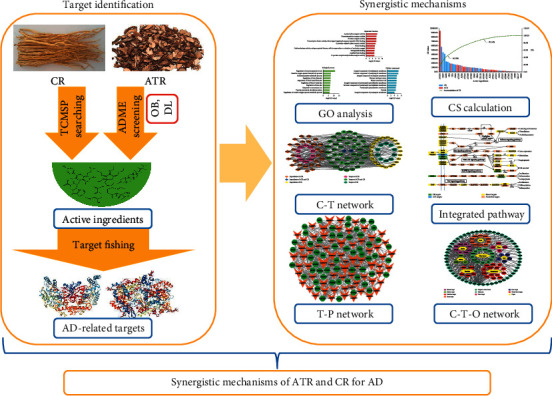
The complete framework of the systems pharmacology approach.

Consequently, we constructed a systems pharmacology model to explore the therapeutic mechanism of ATR and CR for treating AD. First, the active ingredients selected from the database were screened by two ADME parameters to ensure comprehensiveness. Second, a large-scale analysis of targets was performed by target identification to establish the network. Finally, network construction, including compound-target (C-T), target-pathway (T-P), and compound-target-organ (C-T-O) networks, was applied to reveal the underlying mechanism of the combination of ATR and CR on AD in our study (Figure 1).

## 2. Materials and Methods

### 2.1. Chemical Ingredients Database Construction

All of the constituent data of ATR and CR were retrieved from the TCM Systems Pharmacology Database and Analysis Platform (TCMSP, http://lsp.nwu.edu.cn/) [38]. Subsequently, the initial structures of all ingredients were depicted in TIF format using ChemDraw (version 16.0). Additionally, the pharmacology-related properties, including oral bioavailability (OB), drug-likeness (DL), molecular weight (MW), octanol-water partition coefficient predicted by ACD/PhysChem Suite (AlogP), number of acceptor atoms for H-bonds (nHAcc), and number of donor atoms for H-bonds (nHDon), were obtained from TCMSP.

### 2.2. Active Ingredient Screening by ADME

An early pharmacokinetic evaluation is important due to the failures caused by limited pharmacokinetic profiles in modern drug discovery [39]. However, identifying the pharmacokinetic properties by traditional biological experiments is expensive and time-consuming, and *in silico* methods are efficient strategies for subsequent analysis [36, 40]. In our current study, to identify the active ingredients, we used OB and DL as criteria to select components of ATR and CR.

The OB is the key factor for evaluating the ability of drugs to deliver the compound to the systemic circulation by oral administration. Based on 805 structurally defined Western drugs and drug-like molecules, a novel *in silico* system, OBioavail 1.1, was used to calculate the OB values of all ingredients [41], and those ingredients with OB ≥ 30% were preserved for further analysis.

The values of the DL evaluation approach based on the Tanimoto coefficient were calculated using the following equation: *T*(*A*, *B*)=((*A* × *B*)/(|*A*|^2^+|*B*|^2^ − *A* × *B*)). In this equation, *A* represents the molecular descriptors of herbal compounds, and *B* displays the average molecular properties of all compounds in DrugBank [42]. Although DL ≥ 0.18 was a common criterion for ADME screening [43], the number of ingredients of ATR with DL ≥ 0.18 and OB ≥ 30% was only four. Thus, to obtain more details and information about the two herbs, those ingredients with suitable DL ≥ 0.10 were selected as candidates for active ingredients as in a previous study [21, 44]. Additionally, some ingredients with a low OB or DL were supplemented because of their high bioactivity and enrichment through a text-mining method.

### 2.3. Target Collection

Based on the performance of several approaches integrated with chemometrics methods, message collection, and data mining, the related targets of the active ingredients of ATR and CR were searched. First, the targets of active ingredients were obtained from TCMSP. Second, the active ingredients were submitted to various servers, viz., BindingDB database (http://www.bindingdb.org/bind/index.jsp) [45] and STITCH (http://stitch.embl.de/) [46]. Finally, the targets obtained from the databases were input to UniProt (http://www.uniprot.org/) to standardize the names of the targets.

Then, for further elucidation of the role of ATR and CR in AD treatment, targets of AD were obtained from various servers, viz., Therapeutic Target Database (TTD, http://bidd.nus.edu.sg/group/cjttd/) [47], Comparative Toxicogenomics Database (CTD, http://ctdbase.org/) [48], Online Mendelian Inheritance in Man database (OMIM, http://www.omim.org/), PharmGKB (http://www.pharmgkb.org) [49], and DrugBank (https://www.drugbank.ca/) [50]. Finally, the common targets of ATR and AD or CR and AD were selected for further analysis. Importantly, all targets kept for further analysis were only derived from *Homo sapiens*.

### 2.4. Gene Ontology (GO) Analysis and Network Construction

To better decipher the function of the identified targets, we performed GO enrichment analysis for the 62 target proteins by Clue GO, a widely used Cytoscape plugin [51]. The *P* values were corrected with a Bonferroni step-down method.

Then, for elucidation of the underlying molecular mechanism of action of ATR and CR, three corresponding networks were constructed: (1) A compound-target network (C-T network) was constructed to identify the key targets based on the active ingredients of ATR and CR and their corresponding targets. (2) A target-pathway network (T-P network) was built by determining the relationship between targets and pathways that were extracted from KEGG (Kyoto Encyclopedia of Genes and Genomes, http://www.kegg.jp). *P* values were set at 0.05 as the cut-off criterion. (3) The related targets and their tissue types were used in the compound-target-organ network (C-T-O network). The target organ location was determined in the BioGPS bank (http://biogps.org) [52]. All visualized networks were constructed by the open software Cytoscape 3.7.0 (http://www.cytoscape.org/) [53].

### 2.5. Contribution Score Calculation

For further elucidation of the role of each active ingredient of ATR and CR on AD treatment, a contribution score based on the degree listed by Cytoscape 3.7.0 was calculated by the following equation [16]:

(1) $$\begin{array}{c} A_{ij}=\omega_{ei}+\left|\frac{C_{Ai}+C_{Bi}}{C_{Ai}-C_{Bi}}\right|,\\ \omega_{ei}=\frac{C_{\text{edge}}}{T_{\text{edge}}},\\ \text{CS}\left(i\right)=\sum_{ij}^{n}Ci\times \left[A_{ij}\times P_{j}\right], \end{array}$$

where *i* and *j* represent the number of components and proteins, respectively. *C* is the degree of each component, and *P* is the degree of each protein. *C*_*Ai*_ represents the degree of each component only in the CR C-T network, and *C*_*Bi*_ represents the degree of each ingredient only in the ATR C-T network. *ω*_*ei*_ is the ratio of the degree of each ingredient to all ingredients. *A*_*ij*_ is the index of affinity determined from the *ω*_*ei*_ value. All degrees in this equation are calculated by Cytoscape 3.7.0. The contribution score (CS) represents the network contribution of one ingredient and its effectiveness in AD.

## 3. Results

### 3.1. Differences between ATR and CR

A total of 239 ingredients were retrieved in ATR (105) and CR (134) based on searching in a series of public databases. Lignans and volatile oil were the major components in ATR, whereas polysaccharides and volatile oil were the major components in CR. More details about these ingredients are listed in Table S1.

**Figure 2:**
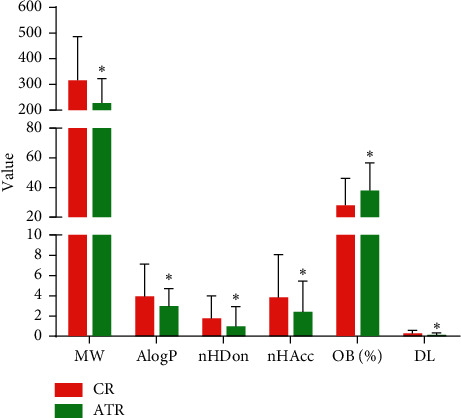
The molecular variation of all ingredients in ATR and CR. ^*∗*^*P* < 0.01 by two tailed *t*-test (vs. CR).

To explore the molecular differences between ATR and CR, we compared six parameters of these components: MW, AlogP, nHDon, nHAcc, OB, and DL. As shown in Figure 2, the values of these components mainly followed Lipinski's rule of five [54]. (1) For MW, the average value of the components in ATR (227.44) was significantly lower than that in CR (316.17) (*P*=3.58*E* − 06). (2) The AlogP values of ATR and CR were 2.99 and 3.96, respectively, demonstrating that both components of these two herbs were hydrotropic. (3) For nHDon and nHAcc, the ATR values (0.99 and 2.42) were all significantly lower than those in CR (1.75 and 3.84) (*P*=6.16*E* − 03,4.10*E* − 03). (4) For OB, unlike the values mentioned above, ATR possessed a higher average OB value (37.97) than CR (27.98) (*P*=4.35*E* − 05), indicating that ATR had better pharmacokinetic properties. (5) For DL, the DL value of ATR (0.147) was significantly lower than that of CR (0.289) (*P*=1.80*E* − 05).

In summary, these data showed a variation between the ingredients of ATR and CR because of their distinct chemicophysical properties. Our results suggested that ATR showed better pharmacokinetic properties, but the ingredients in CR had a better DL. These two herbs contained different main ingredients, which may explain why ATR and CR could produce synergistic and complementary effects.

### 3.2. Identification of Active Ingredients in ATR and CR

The TCM formula usually contains multiple ingredients, but several of them possess unsatisfactory pharmacodynamic and pharmacokinetic properties, influencing the therapeutic responses [55]. Therefore, it is essential to identify the components with favorable properties, and ADME screening is a useful way to select suitable ingredients from herbs or TCM formulas that have many components. In our current work, we employed two major ADME parameters to filter the active ingredients from ATR and CR. As a result, a total of 68 active ingredients from the 239 ingredients were chosen for further analysis (Table 1).

### 3.3. Active Ingredients from ATR

Thirty-three active ingredients were selected from ATR through strict ADME screening rules, and most of them showed ideal biological activities. For instance, kaempferol (OB = 41.88, DL = 0.2) attenuates oxidative stress by regulating Bcl-2 in neuronal cells [56]. Marmesin (OB = 50.28, DL = 0.18) forms hydrogen bonds with paraoxonase to reduce nitric oxide levels to protect rats against myocardial infarction [57]. Cycloartenol (OB = 38.69, DL = 0.78) can reduce oxidase activity [58]. Majudin (OB = 42.21, DL = 0.13) inhibits cholinesterase to increase acetylcholine and butyrylcholine levels [59]. Surprisingly, asarones, accounting for more than 90% of ATR oil, have low DL values [60]. However, *β*-asarone (OB = 35.61, DL = 0.06) and azaron (OB = 38.89, DL = 0.06) could promote neuronal differentiation and reduce intracellular reactive oxygen species accumulation [18, 60]. P-coumaric acid (OB = 43.29, DL = 0.04) can relieve the neuroinflammatory responses induced by amyloid-beta peptide [61]. Vanillic acid exhibits marked antioxidant properties [62]. P-MCA (p-methoxycinnamic acid, OB = 31, DL = 0.05) exerts antihyperglycemic [63] and neuroprotective effects [64]. Bisasaricin (OB = 28.94, DL = 0.5) exhibits antibacterial and anti-inflammatory activity [65]. Above all, a total of 33 active ingredients, including some mentioned above, were preserved for the active ingredients of ATR.

### 3.4. Active Ingredients from CR

Among the 134 ingredients in CR, 36 ingredients were selected by ADME screening. EIC (linoleic acid, OB = 41.90, DL = 0.14) could reduce the development of atherosclerosis, having a strong association with AD and vascular dementia due to its antioxidant and anti-inflammatory effects [66, 67]. Stigmasterol (OB = 43.83, DL = 0.76) has beneficial effects on vascular function by improving the lipoprotein profile [68]. DIOP (OB = 43.59, DL = 0.39) exhibits anti-inflammatory activity based on its cholinesterase inhibitory activity [51]. Evidence proves that syringin (OB = 14.64, DL = 0.32) is an efficient treatment for AD [69]. Luteolin (OB = 36.16, DL = 0.25) depresses the proliferation and migration of vascular smooth muscle cells to prevent atherosclerosis [70]. Hydroxymethylfurfural (HMF; OB = 45.07, DL = 0.02) and apigenin (OB = 23.06, DL = 0.21) were shared by two herbs. HMF was selected because of its therapeutic effect on AD, and it provided effective protection against cognitive impairment via the activation of NMDA receptor signaling [71]. Oral treatment with apigenin plays an important role in the neuroprotective effects by relieving A*β* deposition and improving antioxidative activity [72]. All these components are listed as potential active ingredients for CR, and the details of the 68 ingredients of the two herbs are shown in Table 1.

### 3.5. Target Proteins of ATR and CR

Identifying the targets of candidate ingredients based on experimental approaches is complex and time-consuming. Recently, an integrated *in silico* approach was used to identify the corresponding targets for the active ingredients of ATR and CR. In our study, predictive models, including TCMSP, SEA, and STITCH, were used to search for the targets of ATR and CR, and 218 and 229 proteins were identified as targets of ATR and CR, respectively (Figure S1**)**. Next, these targets were sent to TTD, CTD, OMIM, DrugBank, and PharmGKB to determine whether they were related to AD. In total, for ATR and CR, by target fishing, 53 out of 68 active ingredients (15 active ingredients have no AD-related targets) were valid for binding with 62 AD-related target proteins, and the details of 62 AD-related targets are listed in Table 2.

In ATR, 52 AD-related target proteins were validated to bind with 30 active ingredients. For example, the majority of ingredients in ATR, such as ATR6, ATR19, ATR30, ATR54, and ATR93, exhibited strong activation of the neurotransmitter receptors CHRM1, CHRM2, CHRM2, GABRA1, and GABRA2, modulating neurotransmission dysfunction in the AD brain [73, 74]. Intriguingly, ATR93 also interacted with CYP1A2 and CYP3A, which are major drug-metabolizing enzymes and can prevent cholinergic symptoms such as nausea and vomiting in patients with AD [75, 76]. Furthermore, three active ingredients, ATR3, ATR15, and ATR102, were shown to interact with MAOA, MAOB, and NOS3, which are related to inflammation and play a role in the pathogenesis and symptoms of mental disorders [77, 78].

In CR, 52 AD-related target proteins were validated to bind with 25 active ingredients, including ACHE, APP, BCHE, BCL2, BAX, CASP3, and CASP7, and are implicated in cell apoptosis and vascular and nervous system diseases. For instance, ACHE and BCHE are the major therapeutic targets of AD that improve *β*-amyloid plaques and cholinergic function in AD patients [79, 80]. Remarkably, luteolin (CR63) and glycitein (CR106) interact with APP, the central pathological target of AD [81]. Indeed, luteolin can prevent the formation of *β*-amyloid protein by inhibiting ppGalNAc-T activity [82]. Glycitein can also suppress *β*-amyloid deposition and has antioxidative activity [83]. Additionally, our results found that the apoptosis-related genes BCL2, BAX, CASP3, and CASP7, associated with AD, were potential targets of CR63 and CR77 [84, 85]. All these findings may explain why CR can treat AD with ATR.

### 3.6. Synergistic Mechanisms of ATR and CR

#### 3.6.1. GO Enrichment Analysis for Targets

**Figure 3:**
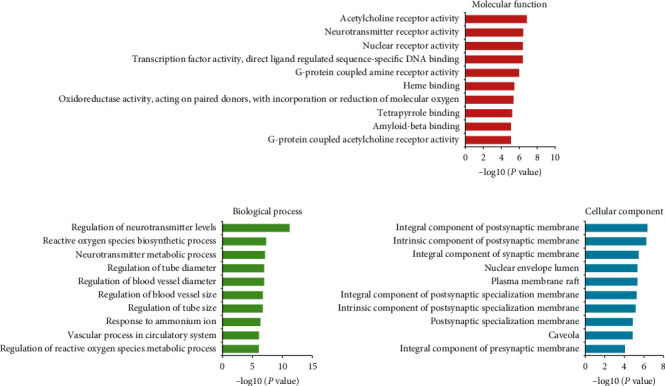
GO enrichment analysis of the targets of ATR and CR.

To clarify the synergistic mechanism of ATR and CR, we performed GO enrichment analysis on Clue Go. In Figure 3, the top 10 significantly enriched terms are ranked in the biological process (BP), molecular function (MF), and cellular component (CC) categories (*P* < 0.05; *P* values are corrected using the Benjamini–Hochberg procedure), including regulation of neurotransmitter levels, reactive oxygen species biosynthesis, and regulation of blood vessel diameter in the pre- and prosynaptic membrane, nuclear envelope lumen, and plasma membrane, to exert anti-AD potential. Particularly, the neurotransmitter imbalance caused by dysfunction of cholinergic neurons contributed to the cognitive deficits associated with AD [86, 87]. Studies have demonstrated that the degeneration of cholinergic neurons gives rise to memory loss in AD patients [88]. In the early stage of AD, the generation of reactive oxygen species is the prominent feature [89]. Oxidative stress is reported to be involved in the disruption of A*β* clearance in the brain, which contributes to the accumulation of A*β* [90]. Abnormal blood vessel diameters reduce blood flow and lead to an inadequate blood supply in the cortex [91, 92]. Undoubtedly, these BP terms are all closely associated with AD. For instance, synaptic dysfunction caused by disruption of neurotransmitter transmission and increased formation of reactive oxygen species in neural cells are the main pathogenic mechanisms of AD [93, 94]. Additionally, the lower cerebral blood flow caused by blood vessel size is a major risk factor for AD [95]. The results of GO enrichment analysis indicated that the combination of ATR and CR treats AD mainly by neuroprotective, antioxidative, and antiatherosclerotic effects.

#### 3.6.2. Compound-Target Network Analysis

**Figure 4:**
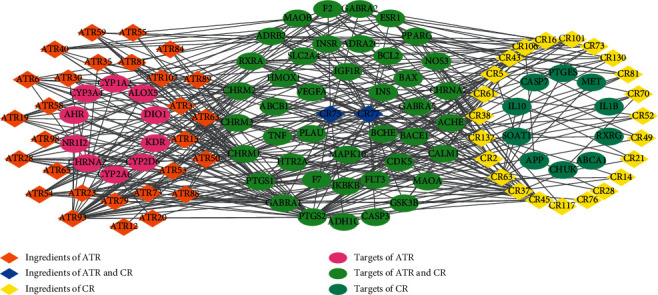
Component-target network of ATR and CR. The orange and yellow rhombus nodes are the active ingredients of ATR and CR, and the pink and light blue ellipse nodes are related targets. The blue rhombus nodes are the shared active ingredients HMF (DS75) and apigenin (DS77). The green ellipse nodes are the shared targets of ATR and CR.

In Figure 4, 53 active ingredients and 62 AD-related targets were used to construct the C-T network. There were 373 component-target associations between 53 active ingredients and 62 AD-related targets. The average number of targets per ingredient was 6.0, and the mean degree of ingredients per target was 7.0, indicating complicated and complementary relationships between ingredients and targets. In total, more than half of the targets (67.7%), such as ACHE, BAX, BCL2, GABRA1, NOS3, and PTGS1, were synergistically regulated by various components of ATR and CR. However, several targets (32.3%), such as APP, CYP1A2, IL10, MAPK10, and VEGFA, were regulated by each ingredient of ATR and CR. However, this Chinese formula focused on the targets associated with AD, such as neurotransmitter release and interaction between neuroactive ligands and receptors (ADRA2C, APP, CASP3, CHRM1, CHRNA7, GABRA1, and CYP2D6). It also modulated other targets involved in the pathogenesis of AD, including oxidative stress, atherosclerosis, and inflammation (BCL2, IKBKB, MAPK10, NOS3, TNF, PTGS2, and GSK3B). As shown in Figure 4, kaempferol (degree = 33) has the highest degree, followed by apigenin (degree = 30), stigmasterol (degree = 16), 7-methoxy-2-methyl isoflavone (degree = 16), luteolin (degree = 15), and 8-isopentenyl-kaempferol (degree = 12), indicating that these ingredients play crucial roles in the treatment of AD. For instance, kaempferol is reported to promote the level of antioxidants and alleviate neuroinflammation in the hippocampus to protect against cognitive deficits in AD [96]. Apigenin can suppress the expression of cytokines and the production of nitric oxide, indicating a potential drug for AD [97]. Stigmasterol could decrease *β*-secretase activity and BACE1 internalization, which is related to APP *β*-secretase cleavage [98]. Luteolin also has several biological functions, including anti-neuroinflammatory and antioxidant activities, which are beneficial for the prevention of AD [99].

### 3.7. Target-Pathway Network Analysis

**Figure 5:**
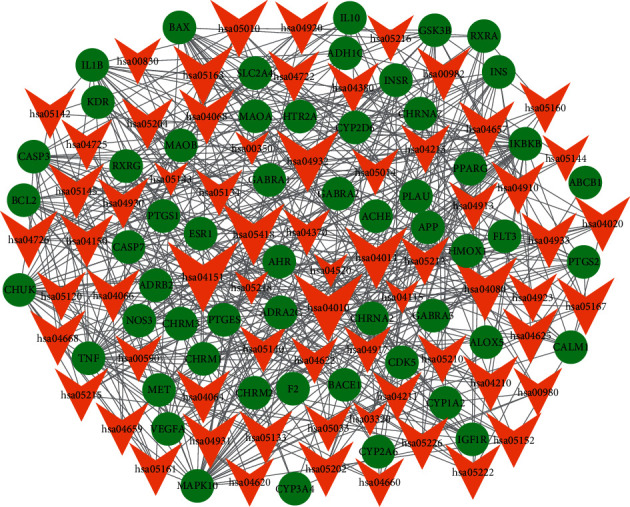
Target-pathway networks of ATR and CR. The green nodes are the targets of ATR and CR, while the orange nodes represent the pathways.

All of the targets interacting with the active ingredients were mapped onto the 67 KEGG pathways, and the T-P network was generated. As shown in Figure 5, the PI3K-Akt signaling pathway shows the highest number of target connections (degree = 15), followed by the MAPK signaling pathway (degree = 13), neuroactive ligand-receptor interaction (degree = 12), fluid shear stress and atherosclerosis (degree = 11), nonalcoholic fatty liver disease (NAFLD) (degree = 11), and Ras signaling pathway (degree = 11). These pathways were related to neurofibromatosis, antiatherosclerosis, antioxidation, and anti-inflammation. The PI3K-Akt signaling pathway is involved in atherosclerosis by blocking blood flow and inhibiting the migration of fibroblasts [9] and can suppress neuronal cell death and improve tau hyperphosphorylation by BCL2, GSK3B, and IGF1R [100–102]. MAPK can directly affect AD, contributing to neuroinflammation and acting in some processes, such as excitotoxicity, synaptic plasticity, and tau phosphorylation [103, 104]. The neuroactive ligand-receptor interaction pathway has been applied in the analysis of neurodegenerative disorders [105]. As expected, our results showed that the combined herbs modulated neuroactive receptors such as CHRM1, CHRM2, GABRA1, GABRA2, etc., which are involved in this pathway and provide therapeutic benefits for AD patients. The fluid shear stress and atherosclerosis pathway can regulate the size of cerebral blood vessels [14] as well as plaque formation, which will lead to vascular dementia [106].

**Figure 6:**
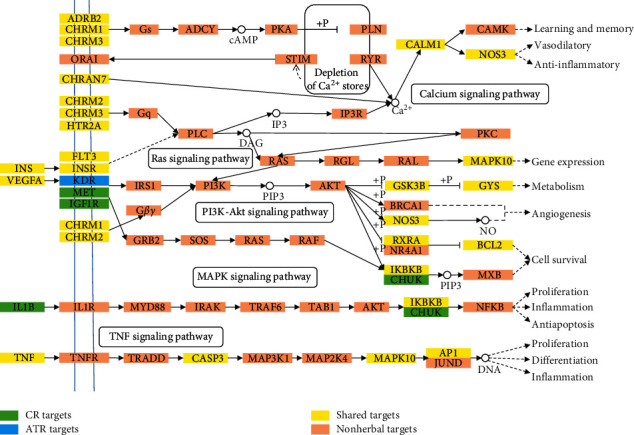
Distribution of partial targets of ATR and CR on the compressed pathway.

The compressed pathway was constructed to further explore the details of the synergetic mechanism of ATR and CR in the treatment of AD. As shown in Figure 6, ATR could act on the KDR gene, which is one of the key molecules related to angiogenesis and a strong risk factor for atherosclerosis [107], while CR can act on its ligand VEGFA. This result indicated that ATR and CR could treat AD through complementary effects on atherosclerosis, which is a pathogenic factor of AD. Additionally, CR can act on the genes of the upstream pathway, such as IL1B and TNF, which are proinflammatory mediators [17, 26], while ATR can act on downstream genes, such as CALM1 and NOS3, which are associated with learning and memory and anti-inflammatory processes. Consequently, these results showed that ATR and CR have a synergistic effect on antiatherosclerosis processes, anti-inflammatory processes, learning, and memory in a comprehensive pathway.

### 3.8. Contribution Score Analysis

**Figure 7:**
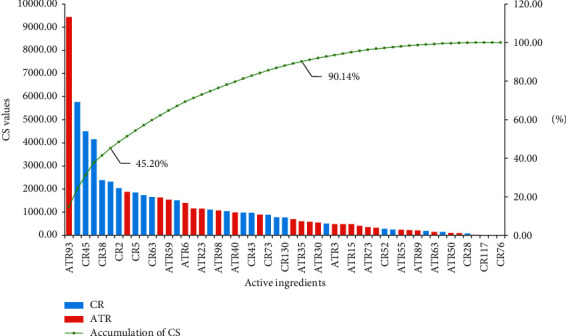
CS and accumulative CS of active ingredients in ATR and CR.

To further explore the synergetic mechanism of ATR and CR, we performed CS analysis following a previously described method [16]. The CS value of each active ingredient is listed in Figure 7 and Table S2. The top 6 ingredients were kaempferol (SCP93), apigenin (DS77), 7-methoxy-2-methyl isoflavone (DS45), stigmasterol (DS37), syringin (DS38), and HMF (DS75) with a CS sum of 45.20%; these were active ingredients from both ATR and CR, especially apigenin and HMF, the shared ingredients. For instance, syringin exhibits anti-inflammatory activity and enhances the synthesis of acetylcholine in the hippocampus [69, 108]. HMF can mitigate the impairment of cognition and memory function induced by A*β*, inhibit beta-secretase activity, and increase antioxidative enzyme activities [109]. Moreover, 29 ingredients could contribute to the effects of this formula on AD with a CS sum of 90.14%, and ATR and CR accounted for 43.13% and 56.87%, respectively (Figure S2). Our data fully explained why the combination of ATR and CR could generate synergistic and combinatorial effects on AD.

### 3.9. Compound-Target-Organ Network Analysis

**Figure 8:**
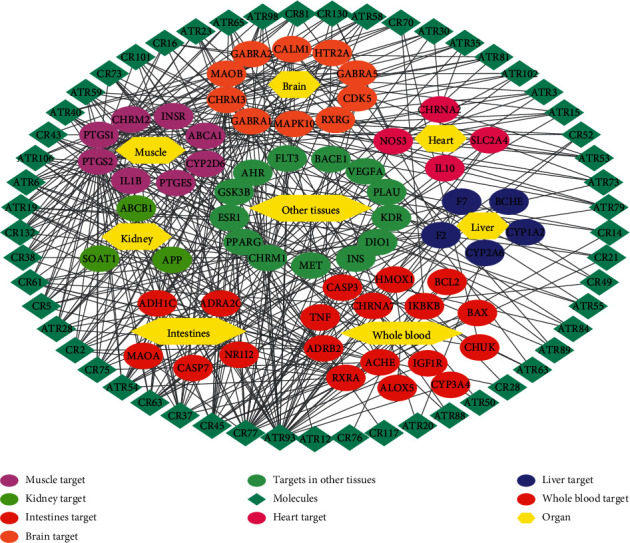
Compound-target-organ networks of ATR and CR.

In TCM, the human body is considered an organic whole, and the organs are complementary to each other. The curative effect of diseases is usually exerted by multiorgan cooperation. Therefore, to better understand the synergistic mechanism of ATR and CR treatment for AD at the organ level, we generated a compound-target-organ network. The mRNA expression results were obtained from the BioGPS database, and the target was positioned in the organization where it had the highest expression patterns. As shown in Figure 8, the targets were widely distributed on the brain, heart, kidney, liver, lung, whole blood, and other tissues. Particularly, the top three distributed organs of targets are whole blood, brain, and muscle. Undoubtedly, the key pathogenic mechanism of AD is the degradation of the brain, and the main targets located in the brain are neurotransmitter receptors such as CHRM3, GABRA1, and GABRA2. As a pivot between each tissue, whole blood improves the coordination of organs to provide a positive effect on AD. Additionally, the gene expression pattern in blood indicates that biological pathways associated with oxidative stress, inflammation, apoptosis, and immune activation are involved in AD [110, 111]. The dysfunction of smooth muscle, which is responsible for regulating blood vessels, could lead to AD complications, such as atherosclerosis and other vascular diseases [22]. In total, these data indicated that the combination of ATR and CR effectively prevents AD not only in the brain but also in other organs. This evidence indicated that ATR and CR have synergistic effects at the organ level as well.

## 4. Discussion

AD is a neurodegenerative disease characterized by synapse loss [112], neurofibrillary tangles in the brain [113], and extracellular deposits of *β*-amyloid peptides (A*β*) [114]. The pathogenesis of AD also involves inflammatory and oxidative stress in neuronal cells [4, 6], coronary heart disease [115, 116], and cardiovascular disease [66]. Our results demonstrated that the combination of ATR and CR can effectively relieve inflammation, oxidative stress, atherosclerosis development, and nervous system damage.

First, 68 active ingredients were chosen following ADME screening of 235 ingredients. In particular, apigenin and HMF were both shared ingredients of the two herbs that had not passed the screening criteria, but they were preserved because of their neuroprotective effects [71, 72]. Moreover, the volatile oils of ATR, such as *β*-asarone and *γ*-asarone, could improve learning and memory, mitigate the deposits of A*β*, and prevent oxidative stress-induced apoptosis [17, 56]. The sterols of CR, such as stigmasterol and spinasterol, could attenuate the risk of coronary heart disease and inhibit the development of atherosclerosis [117, 118]. Consequently, these results suggest that ATR and CR could produce a combinatorial effect on AD.

Second, according to the CS calculation of each ingredient, ATR and CR can contribute to the effects for AD with a sum of 43.13% and 56.87%, respectively, and 29 ingredients of the two herbs can contribute a sum of 90.51%. This result suggested that the therapeutic effect of this formula was derived from ATR and CR, not a single herb nor a handful of ingredients. More remarkably, the top ingredients of the CS in ATR, such as kaempferol, not only could inhibit inflammation [119] but also attenuate glutamate-induced oxidative stress [56]. However, for CR, the top ingredients, such as stigmasterol and syringin, showed strong efficacy and treatment of atherosclerosis [68, 120]. These results suggested that ATR provided neuroprotective effects such as antioxidation and anti-inflammation, and subsequently, CR affected AD by inhibiting the development of atherosclerosis. These data proved evidence that the combination of ATR and CR had synergistic effects on AD.

Third, the GO enrichment analysis showed that the top ten BP terms were all closely related to neurotransmitter transduction, antioxidant effects, and vasodilatation, which are associated with AD in the synaptic membrane and plasma membrane. These data implied that these two herbs displayed an excellent ability to treat AD and its accompanying symptoms. The results of C-T network analysis showed that CR could act on AD-related genes, including APP [120], IL10 [56], CASP7 [68], and SOAT1 [121], and ATR could act on AD-related genes, including CYP2D6 [122], CHRNA2 [123], and ALOX5 [124], indicating that these two herbs displayed a complementary ability to treat AD. Several AD-related genes, such as ACHE [125], PTGS2 [126], IKBKB [127], GABAR1 [128], CHRM2/3 [129], etc., were synergistically regulated by various ingredients of ATR and CR, demonstrating that their combination enhances the ability to treat AD. Furthermore, based on the T-P network analysis, ATR and CR treatment of AD mainly depends on the PI3K signaling pathway, MAPK signaling pathway, neuroactive ligand–receptor interaction, and fluid shear stress and atherosclerosis, which regulate the nervous and vascular systems. Additionally, this combination activates nonalcoholic fatty liver disease, the Ras signaling pathway, and human cytomegalovirus infection to relieve the AD-associated symptoms such as liver inflammation and cytomegalovirus infection to benefit patients with AD [6, 68].

Finally, the integrated pathway analysis showed that ATR and CR could act on genes that were involved in various signaling pathways. In particular, KDR was a unique target of ATR, while CR could act on its ligand VEGFA, suggesting a complementary effect. Subsequently, CR could act on IGF1R, MET, IL1B, and CHUK alone. These results provided powerful evidence that each of them could act on genes to activate the complementary signaling pathway for treating AD. The C-T-O network analysis also showed that the targets of ATR and CR had a wide distribution, but the top 3 organs were whole blood, brain, and muscle, which are strongly related to antioxidant, anti-inflammatory, antiatherosclerotic, and neuroprotective activities. Consequently, the synergistic effect of ATR and CR involved not only the molecular and pathway levels but also the organ level.

In summary, this novel systems pharmacology model provided a powerful method to explore the therapeutic mechanism of TCM, which has multiple ingredients and targets. A combination of compatible remedies for nervous system diseases can relieve the symptoms of cardiovascular diseases and oxidative stress, which are the pathogenic mechanisms of AD at different levels. Given the above factors, a mixture of ATR and CR possesses synergism on AD and this study provides a solid theoretical basis for the effective prevention and treatment of the pain caused by AD. However, this conclusion mainly depends on model prediction and literature analysis, and more experiments should be performed to verify our conclusion.

### 4.1. Contribution to the Field Statements

AD is a progressive neurodegenerative disorder caused by extensive synapse loss, the formation of intracellular neurofibrillary tangles, extracellular deposits of *β*-amyloid peptides (A*β*), and inflammatory and oxidative stress in neurons. It has been reported that more than 35 million people suffer from AD. In this work, we evaluated the systems pharmacology-based strategy to explore the pathogenic mechanism of AD at the molecular pathway and organ levels. We first clarified the synergistic and complementary mechanisms of the Chinese herbs ATR and CR in the treatment of AD without laborious and time-consuming experiments. In view of the rich natural compounds in Chinese herbs, this combination will provide an effective treatment for AD.

## Figures and Tables

**Table 1:** Information on the active ingredients in ATR and CR.

ID	Molecule name	Structure	OB (%)	DL
CR1	Poriferasta-7,22E-dien-3beta-ol	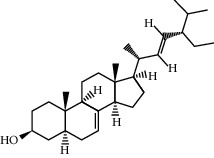	42.98	0.76
CR2	2-methoxyfuranodiene	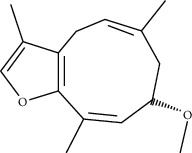	53.58	0.13
CR5	EIC	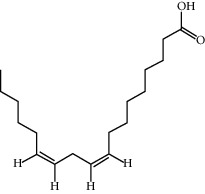	41.9	0.14
CR14	Methyl linoleate	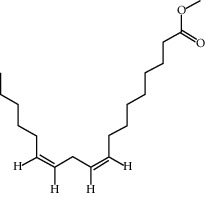	41.93	0.17
CR16	(+/−)-Isoborneol	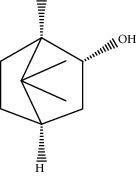	86.98	0.05
CR21	Perlolyrine	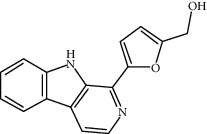	65.95	0.27
CR28	DIOP	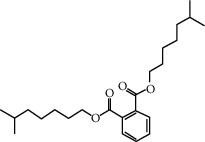	43.59	0.39
CR32	ZINC03978781	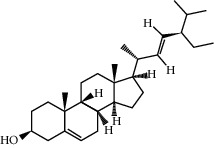	43.83	0.76
CR37	Stigmasterol	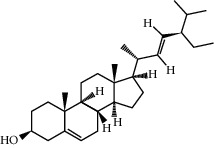	43.83	0.76
CR38	Syringin	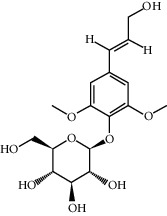	14.64	0.32
CR43	Tectorigenin	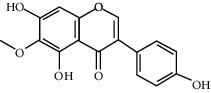	28.41	0.27
CR45	7-Methoxy-2-methyl isoflavone	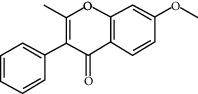	42.56	0.2
CR48	Spinasterol	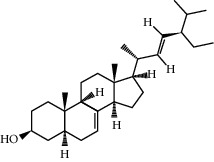	42.98	0.76
CR49	Atractylenolide II	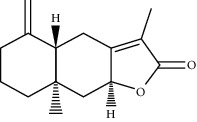	47.5	0.15
CR52	Atractylenolide III	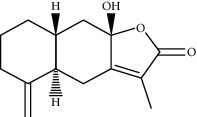	68.11	0.17
CR61	Frutinone A	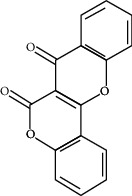	65.9	0.34
CR63	Luteolin	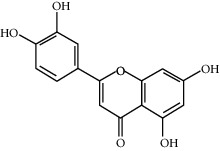	36.16	0.25
CR67	Taraxerol	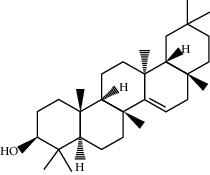	38.4	0.77
CR69	Stigmast-7-enol	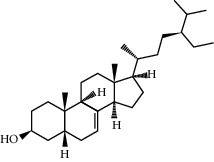	37.42	0.75
CR70	Norharman	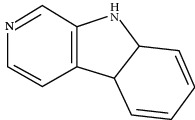	18.88	0.08
CR73	3-Beta-hydroxymethyllenetanshiquinone	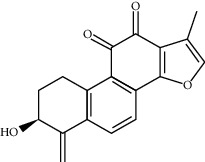	32.16	0.41
CR75	HMF	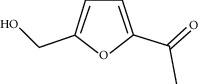	45.07	0.02
CR76	Methyl icosa-11,14-dienoate	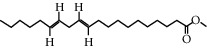	39.67	0.23
CR77	Apigenin	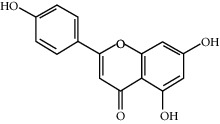	23.06	0.21
CR81	(1R)-2,3,4,9-Tetrahydro-1H-pyrido[3,4-b]indol-2-ium-1-carboxylate	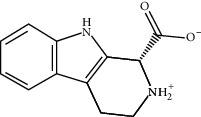	52.9	0.13
CR97	5-Alpha-stigmastan-3,6-dione	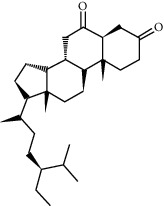	33.12	0.79
CR99	7-(Beta-xylosyl)cephalomannine_qt	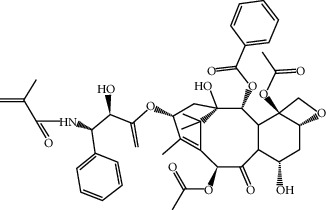	38.33	0.29
CR101	Codonopsine	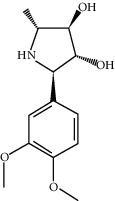	45.83	0.13
CR103	Daturilin	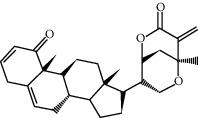	50.37	0.77
CR106	Glycitein	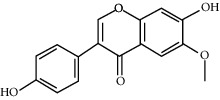	50.48	0.24
CR112	Spinoside A	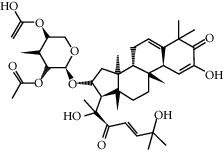	39.97	0.4
CR113	(8S,9S,10R,13R,14S,17R)-17-[(E,2R,5S)-5-ethyl-6-methylhept-3-en-2-yl]-10,13-dimethyl-1,2,4,7,8,9,11,12,14,15,16,17-dodecahydrocyclopenta[a]phenanthren-3-one	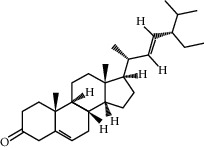	45.4	0.76
CR117	11-Hydroxyrankinidine	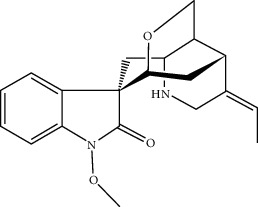	40	0.66
CR123	Ethyl-*β*-D-fructofuranoside	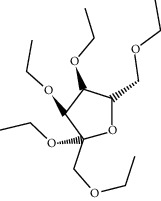	33.84	0.15
CR130	Furanodiene	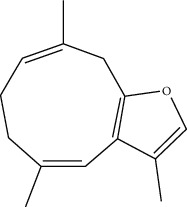	45.11	0.1
CR132	(+)-Beta-pinene	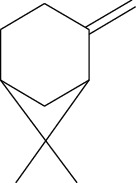	44.77	0.05
ATR3	Vanillic acid	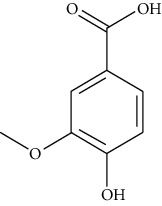	35.47	0.04
ATR6	(−)-Alloaromadendrene	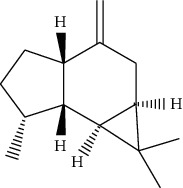	54.04	0.1
ATR12	Calarene	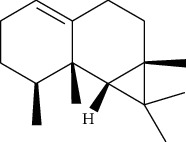	52.16	0.11
ATR15	p-MCA	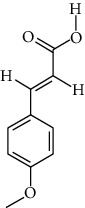	31	0.05
ATR19	Marmesin	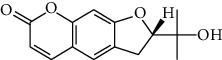	50.28	0.18
ATR20	Majudin	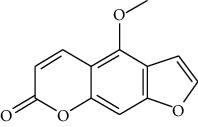	42.21	0.13
ATR23	(−)-Caryophyllene oxide	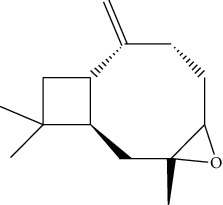	32.67	0.13
ATR28	beta-Asarone	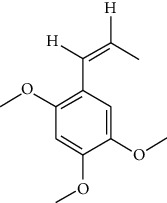	35.61	0.06
ATR30	beta-Gurjunene	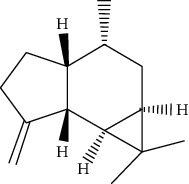	51.36	0.1
ATR35	beta-Cubebene	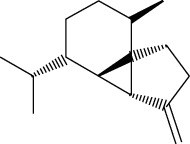	32.81	0.11
ATR40	2′-0.1Methylisoliquiritigenin	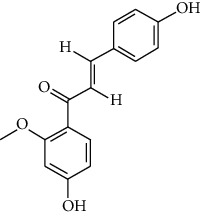	75.86	0.17
ATR50	(+)-Ledene	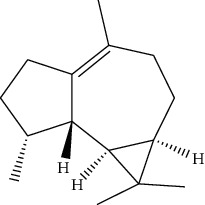	51.84	0.1
ATR53	(+)-alpha-Longipinene	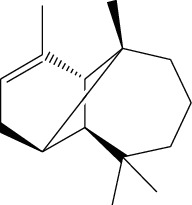	57.47	0.12
ATR54	8-Isopentenyl-kaempferol	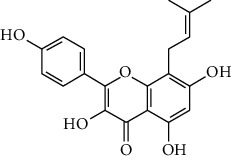	38.04	0.39
ATR55	Aminacrine	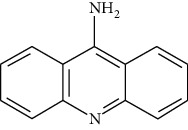	35	0.12
ATR57	Aristolene	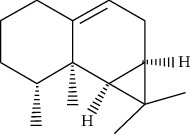	52.2	0.11
ATR58	Aristolone	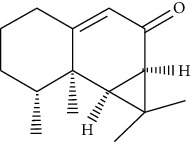	45.31	0.13
ATR59	Azaron	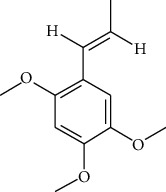	38.39	0.06
ATR63	Bisasarcin	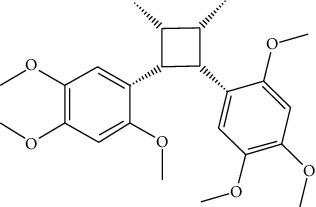	18.55	0.5
ATR65	Calamendiol	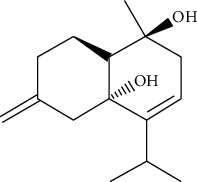	61.13	0.11
ATR73	Isocalamendiol	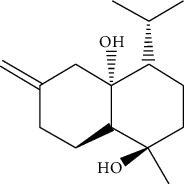	57.63	0.11
ATR78	Longicyclene	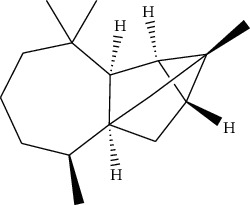	46.07	0.15
ATR79	Murolan-3,9(11)-diene-10-peroxy	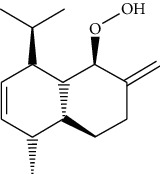	36.72	0.11
ATR81	Patchoulene	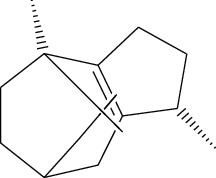	49.06	0.11
ATR84	Spathulenol	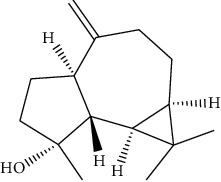	81.61	0.12
ATR87	*α*-Gurjunene	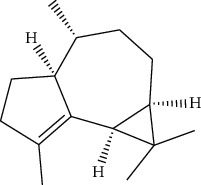	52.57	0.1
ATR88	*α*-Panasinsene	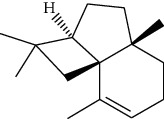	56.77	0.12
ATR89	(1R,3aS,4R,6aS)-1,4-bis(3,4-dimethoxyphenyl)-1,3,3a,4,6,6a-hexahydrofuro[4,3-c]furan	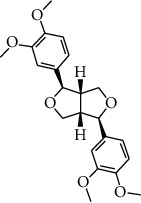	52.35	0.62
ATR91	Cycloartenol	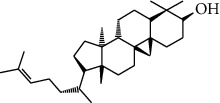	38.69	0.78
ATR93	Kaempferol	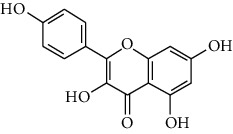	41.88	0.24
ATR98	(−)-alpha-Cedrene	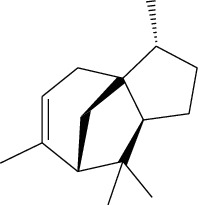	55.56	0.1
ATR102	p-Coumaric acid	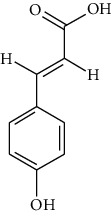	43.29	0.04

**Table 2:** Information on AD-related targets.

Gene	Target name	UniProt ID
ABCA1	ATP-binding cassette subfamily A member 1	O95477
ABCB1	Multidrug resistance protein 1	P08183
ACHE	Acetylcholinesterase	P22303
ADH1C	Alcohol dehydrogenase 1C	P00326
ADRA2C	Alpha-2C adrenergic receptor	P18825
ADRB2	Beta-2 adrenergic receptor	P07550
AHR	Aryl hydrocarbon receptor	P35869
ALOX5	Arachidonate 5-lipoxygenase	P09917
APP	Amyloid beta A4 protein	P05067
BACE1	Beta-secretase 1	P56817
BAX	Apoptosis regulator BAX	Q07812
BCHE	Cholinesterase	P06276
BCL2	Apoptosis regulator Bcl-2	P10415
CALM1	Calmodulin	P0DP23
CASP3	Caspase-3	P42574
CASP7	Caspase-7	P55210
CDK5	Cyclin-dependent kinase 5 (CDK5)	Q00535
CHRM1	Muscarinic acetylcholine receptor M1	P11229
CHRM2	Muscarinic acetylcholine receptor M2	P08172
CHRM3	Muscarinic acetylcholine receptor M3	P20309
CHRNA2	Neuronal acetylcholine receptor subunit alpha-2	Q15822
CHRNA7	Neuronal acetylcholine receptor protein, alpha-7 chain	P36544
CHUK	Inhibitor of NF-kappa-B kinase (IKK)	O15111
CYP1A2	Cytochrome P450 1A2	P05177
CYP2A6	Cytochrome P450 2A6	P11509
CYP2D6	Cytochrome P450 2D6 (2D6)	P10635
CYP3A4	Cytochrome P450 3A4	P08684
DIO1	Type I iodothyronine deiodinase	P49895
ESR1	Estrogen receptor	P03372
F2	Thrombin	P00734
F7	Coagulation factor VII	P08709
FLT3	Tyrosine-protein kinase receptor FLT3	P36888
GABRA1	Gamma-aminobutyric acid receptor subunit alpha-1	P14867
GABRA2	Gamma-aminobutyric-acid receptor alpha-2 subunit	P47869
GABRA5	Gamma-aminobutyric-acid receptor alpha-5 subunit	P31644
GSK3B	Glycogen synthase kinase-3 beta	P49841
HMOX1	Heme oxygenase 1	P09601
HTR2A	5-Hydroxytryptamine 2A receptor	P28223
IGF1R	Insulin-like growth factor 1 receptor	P08069
IKBKB	Inhibitor of nuclear factor kappa-B kinase subunit beta	O14920
IL10	Interleukin-10	P22301
IL1B	Interleukin-1 beta	P01584
INS	Insulin	P01308
INSR	Insulin receptor	P06213
KDR	Vascular endothelial growth factor receptor 2	P35968
MAOA	Amine oxidase [flavin-containing] A	P21397
MAOB	Amine oxidase [flavin-containing] B	P27338
MAPK10	Mitogen-activated protein kinase 10	P53779
MET	Hepatocyte growth factor receptor	P08581
NOS3	Nitric-oxide synthase, endothelial	P29474
NR1I2	Nuclear receptor subfamily 1 group I member 2	O75469
PLAU	Urokinase-type plasminogen activator	P00749
PPARG	Peroxisome proliferator activated receptor gamma	P37231
PTGES	Prostaglandin E synthase	O14684
PTGS1	Prostaglandin G/H synthase 1	P23219
PTGS2	Prostaglandin G/H synthase 2	P35354
RXRA	Retinoic acid receptor RXR-alpha	P19793
RXRG	Retinoic acid receptor RXR-gamma	P48443
SLC2A4	Solute carrier family 2, facilitated glucose transporter member 4	P14672
SOAT1	Acyl-cholesterol acyltransferase 1	P35610
TNF	Tumor necrosis factor	P01375
VEGFA	Vascular endothelial growth factor A	P15692

## Data Availability

The data used to support the findings of this study are available from the corresponding author upon request.

## Supplementary Materials

Supplementary Materials
*Figure S1*. The number of targets of ATR (red), CR (blue), and Alzheimer's disease (green) obtained from databases. *Figure S2*. CS and accumulative CS of the active ingredients in ATR or CR. *Table S1*. Detailed information on the ingredients in ATR and CR. *Table S2*. The value of active ingredients in ATR and CR.

## References

[1] Batsh N. L., Mittelman M. S. (2012). *World Alzheimer Report 2012: Overcoming the Stigma of Dementia*.

[2] Acosta, D., A. Ketteringham, and C. Ballard, Alzheimer’s Disease International, 2012

[3] Wang Z.-Y., Liu J.-G., Li H., Yang H.-M. (2016). Pharmacological effects of active components of Chinese herbal medicine in the treatment of Alzheimer’s disease: a review. *The American Journal of Chinese Medicine*.

[4] Kovacic P., Somanathan R. (2012). Redox processes in neurodegenerative disease involving reactive oxygen species. *Current Neuropharmacology*.

[5] Misra S., Medhi B. (2013). Drug development status for Alzheimer’s disease: present scenario. *Neurological Sciences*.

[6] Szaingurten-Solodkin I., Hadad N., Levy R. (2009). Regulatory role of cytosolic phospholipase A2*α* in NADPH oxidase activity and in inducible nitric oxide synthase induction by aggregated A*β*1-42 in microglia. *Glia*.

[7] Hofman A., Ott A., Breteler M. M. (1997). Atherosclerosis, apolipoprotein E, and prevalence of dementia and Alzheimer’s disease in the Rotterdam study. *The Lancet*.

[8] Arvanitakis Z., Capuano A. W., Leurgans S. E., Bennett D. A., Schneider J. A. (2016). Relation of cerebral vessel disease to Alzheimer’s disease dementia and cognitive function in elderly people: a cross-sectional study. *The Lancet Neurology*.

[9] Bettens K., Sleegers K., Van Broeckhoven C. (2010). Current status on Alzheimer disease molecular genetics: from past, to present, to future. *Human Molecular Genetics*.

[10] Benjamin B., Burns A. (2007). Donepezil for Alzheimer’s disease. *Expert Review of Neurotherapeutics*.

[11] Tayeb H. O., Yang H. D., Price B. H., Tarazi F. I. (2012). Pharmacotherapies for Alzheimer’s disease: beyond cholinesterase inhibitors. *Pharmacology & Therapeutics*.

[12] Silva T., Reis J., Teixeira J., Borges F. (2014). Alzheimer’s disease, enzyme targets and drug discovery struggles: from natural products to drug prototypes. *Ageing Research Reviews*.

[13] Kumar A., Singh A., Ekavali (2015). A review on Alzheimer’s disease pathophysiology and its management: an update. *Pharmacological Reports*.

[14] Luo J., Shang Q., Han M., Chen K., Xu H. (2014). Traditional Chinese medicine injection for angina pectoris: an overview of systematic reviews. *The American Journal of Chinese Medicine*.

[15] Su Y., Wang Q., Wang C., Chan K., Sun Y., Kuang H. (2014). The treatment of Alzheimer’s disease using Chinese medicinal plants: from disease models to potential clinical applications. *Journal of Ethnopharmacology*.

[16] Wang C. (2018). System pharmacology-based strategy to decode the synergistic mechanism of Zhi-Zhu Wan for functional dyspepsia. *Frontiers in Pharmacology*.

[17] Mao J., Huang S., Liu S. (2015). A herbal medicine for Alzheimer’s disease and its active constituents promote neural progenitor proliferation. *Aging Cell*.

[18] Lam K. Y. C., Yao P., Wang H., Duan R., Dong T. T. X., Tsim K. W. K. (2017). Asarone from Acori Tatarinowii rhizome prevents oxidative stress-induced cell injury in cultured astrocytes: a signaling triggered by Akt activation. *PLoS One*.

[19] Agathocleous M., Iordanova I., Willardsen M. I. (2009). A directional Wnt/-catenin-Sox2-proneural pathway regulates the transition from proliferation to differentiation in the Xenopus retina. *Development*.

[20] Zhong Y. (2006). *Dictionary of Chinese Traditional Medicine*.

[21] Chan J. Y.-W., Lam F.-C., Leung P.-C., Che C.-T., Fung K.-P. (2009). Antihyperglycemic and antioxidative effects of a herbal formulation of radix astragali, radix codonopsis and cortex lyciiin a mouse model of type 2 diabetes mellitus. *Phytotherapy Research*.

[22] Cai Y. M., Zhang Y., Zhang P. B. (2016). Neuroprotective effect of Shenqi Fuzheng injection pretreatment in aged rats with cerebral ischemia/reperfusion injury. *Neural Regeneration Research*.

[23] Lin S. M., Wang J., Zhou R. Q., Yu Z. H. (2003). Clinical study on treatment of Alzheimer’s disease from the viewpoint of Xin and Shen in Chinese. *Chinese Journal of Integrative Medicine*.

[24] Pan W. (2014). Shen-Zhi-Ling oral liquid improves behavioral and psychological symptoms of dementia in Alzheimer’s disease. *Evidence-Based Complementary and Alternative Medicine*.

[25] Kiyohara H., Matsumoto T., Yamada H. (2004). Combination effects of herbs in a multi-herbal formula: expression of Juzen-taiho-to’s immuno-modulatory activity on the intestinal immune system. *Evidence-Based Complementary and Alternative Medicine*.

[26] Tsai S.-J., Lin C.-Y., Mong M.-C., Ho M.-W., Yin M.-C. (2010). s-Ethyl cysteine ands-propyl cysteine alleviate *β*-amyloid induced cytotoxicity in nerve growth factor differentiated PC12 cells. *Journal of Agricultural and Food Chemistry*.

[27] Guo Q., Zhong M., Xu H., Mao X., Zhang Y., Lin N. (2015). A systems biology perspective on the molecular mechanisms underlying the therapeutic effects of Buyang Huanwu decoction on ischemic stroke. *Rejuvenation Research*.

[28] Zhou W., Wang Y., Lu A., Zhang G. (2016). Systems pharmacology in small molecular drug discovery. *International Journal of Molecular Sciences*.

[29] Zhou W., Wang Y. (2014). A network-based analysis of the types of coronary artery disease from traditional Chinese medicine perspective: potential for therapeutics and drug discovery. *Journal of Ethnopharmacology*.

[30] Liu J. (2016). Systems-pharmacology dissection of traditional Chinese medicine compound saffron formula reveals multi-scale treatment strategy for cardiovascular diseases. *Scientific Reports*.

[31] Zhou W. (2016). Systems pharmacology exploration of botanic drug pairs reveals the mechanism for treating different diseases. *Scientific Reports*.

[32] Huang C., Zheng C., Li Y., Wang Y., Lu A., Yang L. (2014). Systems pharmacology in drug discovery and therapeutic insight for herbal medicines. *Briefings in Bioinformatics*.

[33] Zhang W. (2016). Systems pharmacology dissection of the integrated treatment for cardiovascular and gastrointestinal disorders by traditional Chinese medicine. *Scientific Reports*.

[34] Zheng C., Pei T., Huang C. (2016). A novel systems pharmacology platform to dissect action mechanisms of traditional Chinese medicines for bovine viral diarrhea disease. *European Journal of Pharmaceutical Sciences*.

[35] Lu D., Liu L., Ji X. (2015). The phosphatase DUSP2 controls the activity of the transcription activator STAT3 and regulates TH17 differentiation. *Nature Immunology*.

[36] Yun K.-J., Koh D.-J., Kim S.-H. (2008). Anti-inflammatory effects of sinapic acid through the suppression of inducible nitric oxide synthase, cyclooxygase-2, and proinflammatory cytokines expressions via nuclear factor-*κ*b inactivation. *Journal of Agricultural and Food Chemistry*.

[37] Gu P., Zhu L., Liu Y., Zhang L., Liu J., Shen H. (2017). Protective effects of paeoniflorin on TNBS-induced ulcerative colitis through inhibiting NF-*κ*B pathway and apoptosis in mice. *International Immunopharmacology*.

[38] Ru J. (2014). TCMSP: a database of systems pharmacology for drug discovery from herbal medicines. *Journal of Cheminformatics*.

[39] Rogler G. (2014). Chronic ulcerative colitis and colorectal cancer. *Cancer Letters*.

[40] Dirisina R., Katzman R. B., Goretsky T. (2011). p53 and PUMA independently regulate apoptosis of intestinal epithelial cells in patients and mice with colitis. *Gastroenterology*.

[41] Xu X., Zhang W., Huang C. (2012). A novel chemometric method for the prediction of human oral bioavailability. *International Journal of Molecular Sciences*.

[42] Ma C., Wang L., Xie X.-Q. (2011). GPU accelerated chemical similarity calculation for compound library comparison. *Journal of Chemical Information and Modeling*.

[43] Tao W., Xu X., Wang X. (2013). Network pharmacology-based prediction of the active ingredients and potential targets of Chinese herbal radix curcumae formula for application to cardiovascular disease. *Journal of Ethnopharmacology*.

[44] Song Z., Yin F., Xiang B., Lan B., Cheng S. (2018). Systems pharmacological approach to investigate the mechanism of acori tatarinowii rhizoma for Alzheimer’s disease. *Evidence-Based Complementary and Alternative Medicine*.

[45] Gilson M. K., Liu T., Baitaluk M., Nicola G., Hwang L., Chong J. (2016). BindingDB in 2015: a public database for medicinal chemistry, computational chemistry and systems pharmacology. *Nucleic Acids Research*.

[46] Kuhn M., Szklarczyk D., Franceschini A., von Mering C., Jensen L. J., Bork P. (2012). STITCH 3: zooming in on protein-chemical interactions. *Nucleic Acids Research*.

[47] Li Y. H., Yu C. Y., Li X. X. (2018). Therapeutic target database update 2018: enriched resource for facilitating bench-to-clinic research of targeted therapeutics. *Nucleic Acids Research*.

[48] Davis A. P., Grondin C. J., Johnson R. J. (2019). The comparative toxicogenomics database: update 2019. *Nucleic Acids Research*.

[49] Whirl-Carrillo M., McDonagh E. M., Hebert J. M. (2012). Pharmacogenomics knowledge for personalized medicine. *Clinical Pharmacology & Therapeutics*.

[50] Wishart D. S., Feunang Y. D., Guo A. C. (2018). DrugBank 5.0: a major update to the DrugBank database for 2018. *Nucleic Acids Research*.

[51] Syad A. N., Shunmugiah K. P., Kasi P. D. (2013). Antioxidant and anti-cholinesterase activity of Sargassum wightii. *Pharmaceutical Biology*.

[52] Wu C., Orozco C., Boyer J. (2009). BioGPS: an extensible and customizable portal for querying and organizing gene annotation resources. *Genome Biology*.

[53] Shannon P. (2003). Cytoscape: a software environment for integrated models of biomolecular interaction networks. *Genome Research*.

[54] Zhu K. Y., Xu S. L., Choi R. C.-Y., Yan A. L., Dong T. T.-X., Tsim K. W.-K. (2013). Kai-Xin-San, a Chinese herbal decoction containing ginseng radix et rhizoma, polygalae radix, acori tatarinowii rhizoma, and poria, stimulates the expression and secretion of neurotrophic factors in cultured astrocytes. *Evidence-Based Complementary and Alternative Medicine*.

[55] Lu T., Yang J., Gao X. (2008). Plasma and urinary tanshinol from Salvia miltiorrhiza (Danshen) can be used as pharmacokinetic markers for cardiotonic pills, a cardiovascular herbal medicine. *Drug Metabolism and Disposition*.

[56] Günaltay S. (2014). Differential expression of interleukin-1/toll-like receptor signaling regulators in microscopic and ulcerative colitis. *World Journal of Gastroenterology*.

[57] Krushna G. S., Shivaranjani V. L., Umamaheswari J. (2017). In vivo and molecular docking studies using whole extract and phytocompounds of Aegle marmelos fruit protective effects against isoproterenol-induced myocardial infarction in rats. *Biomedicine & Pharmacotherapy*.

[58] Sultana S., Alam A., Khan N., Sharma S. (2003). Inhibition of benzoyl peroxide and ultraviolet-B radiation induced oxidative stress and tumor promotion markers by cycloartenol in murine skin. *Redox Report*.

[59] Senol F. S., Skalicka Woźniak K., Khan M. T. H., Erdogan Orhan I., Sener B., Głowniak K. (2011). An in vitro and in silico approach to cholinesterase inhibitory and antioxidant effects of the methanol extract, furanocoumarin fraction, and major coumarins of Angelica officinalis L. fruits. *Phytochemistry Letters*.

[60] Lam K. Y. (2016). Asarone from acori tatarinowii rhizoma potentiates the nerve growth factor-induced neuronal differentiation in cultured PC12 cells: a signaling mediated by protein kinase A. *PLoS One*.

[61] Yoon J.-H., Youn K., Ho C.-T., Karwe M. V., Jeong W.-S., Jun M. (2014). p-Coumaric acid and ursolic acid from corni fructus attenuated *β*-amyloid25-35-induced toxicity through regulation of the NF-*κ*B signaling pathway in PC12 cells. *Journal of Agricultural and Food Chemistry*.

[62] Panzella L., Eidenberger T., Napolitano A. (2018). Anti-amyloid aggregation activity of black sesame pigment: toward a novel Alzheimer’s disease preventive agent. *Molecules*.

[63] Adisakwattana S., Roengsamran S., Hsu W. H., Yibchok-anun S. (2005). Mechanisms of antihyperglycemic effect of p-methoxycinnamic acid in normal and streptozotocin-induced diabetic rats. *Life Sciences*.

[64] Kim S. R., Sung S. H., Jang Y. P., Markelonis G. J., Oh T. H., Kim Y. C. (2002). E-p-methoxycinnamic acid protects cultured neuronal cells against neurotoxicity induced by glutamate. *British Journal of Pharmacology*.

[65] Mazlan R. N. A. R. (2018). Solvent extraction and identification of active anticariogenic metabolites in piper cubeba L. through H-1-NMR-Based metabolomics approach. *Molecules*.

[66] Wolozin B., Bednar M. M. (2006). Interventions for heart disease and their effects on Alzheimer’s disease. *Neurological Research*.

[67] Sato M., Shibata K., Nomura R., Kawamoto D., Nagamine R., Imaizumi K. (2005). Linoleic acid-rich fats reduce atherosclerosis development beyond its oxidative and inflammatory stress-increasing effect in apolipoprotein E-deficient mice in comparison with saturated fatty acid-rich fats. *British Journal of Nutrition*.

[68] Liang Y. T., Wong W. T., Guan L. (2011). Effect of phytosterols and their oxidation products on lipoprotein profiles and vascular function in hamster fed a high cholesterol diet. *Atherosclerosis*.

[69] Huang D., Hu Z., Yu Z. (2013). Eleutheroside B or E enhances learning and memory in experimentally aged rats. *Neural Regeneration Research*.

[70] Kordjazy N., Haj-Mirzaian A., Haj-Mirzaian A. (2018). Role of toll-like receptors in inflammatory bowel disease. *Pharmacological Research*.

[71] Lee Y., Gao Q., Kim E. (2015). Pretreatment with 5-hydroxymethyl-2-furaldehyde blocks scopolamine-induced learning deficit in contextual and spatial memory in male mice. *Pharmacology Biochemistry and Behavior*.

[72] Zhao L., Wang J.-L., Liu R., Li X.-X., Li J.-F., Zhang L. (2013). Neuroprotective, anti-amyloidogenic and neurotrophic effects of apigenin in an Alzheimer’s disease mouse model. *Molecules*.

[73] Singh A. K., Kashyap M. P., Tripathi V. K., Singh S., Garg G., Rizvi S. I. (2017). Neuroprotection through rapamycin-induced activation of autophagy and PI3K/Akt1/mTOR/CREB signaling against amyloid-*β*-induced oxidative stress, synaptic/neurotransmission dysfunction, and neurodegeneration in adult rats. *Molecular Neurobiology*.

[74] Guzman B. C. F. (2018). The GABAergic system as a therapeutic target for Alzheimer’s disease. *Journal of Neurochemistry*.

[75] Fontana R. J., Turgeon D. K., Woolf T. F., Knapp M. J., Foster N. L., Watkins P. B. (1996). The caffeine breath test does not identify patients susceptible to tacrine hepatotoxicity. *Hepatology*.

[76] Fontana R. J., deVries T. M., Woolf T. F. (1998). Caffeine based measures of CYP1A2 activity correlate with oral clearance of tacrine in patients with Alzheimer’s disease. *British Journal of Clinical Pharmacology*.

[77] Fan M., Liu B., Jiang T., Jiang X., Zhao H., Zhang J. (2010). Meta-analysis of the association between the monoamine oxidase-A gene and mood disorders. *Psychiatric Genetics*.

[78] Chakrabarti S., Chan C. K., Jiang Y., Davidge S. T. (2012). Neuronal nitric oxide synthase regulates endothelial inflammation. *Journal of Leukocyte Biology*.

[79] Zhao X.-j., Gong D.-m., Jiang Y.-r., Guo D., Zhu Y., Deng Y.-c. (2017). Multipotent AChE and BACE-1 inhibitors for the treatment of Alzheimer’s disease: design, synthesis and bio-analysis of 7-amino-1,4-dihydro-2 H -isoquilin-3-one derivates. *European Journal of Medicinal Chemistry*.

[80] Darvesh S. (2016). Butyrylcholinesterase as a diagnostic and therapeutic target for Alzheimer’s disease. *Current Alzheimer Research*.

[81] Jonsson T., Atwal J. K., Steinberg S. (2012). A mutation in APP protects against Alzheimer’s disease and age-related cognitive decline. *Nature*.

[82] Liu F., Xu K., Xu Z. (2017). The small molecule luteolin inhibits N-acetyl-*α*-galactosaminyltransferases and reduces mucin-type O-glycosylation of amyloid precursor protein. *Journal of Biological Chemistry*.

[83] Gutierrez-Zepeda A. (2005). Soy isoflavone glycitein protects against beta amyloid-induced toxicity and oxidative stress in transgenic Caenorhabditis elegans. *BMC Neuroscience*.

[84] Hu H. Y. (2016). Effect of Qingxinkaiqiao compound on cortical mRNA expression of the apoptosis-related genes Bcl-2, BAX, caspase-3, and A beta in an Alzheimer’s disease rat model. *Journal of Traditional Chinese Medicine*.

[85] Zhang X. L. (2019). A rare missense variant of CASP7 is associated with familial late-onset Alzheimer’s disease. *Alzheimers & Dementia*.

[86] Snowden S. G., Ebshiana A. A., Hye A. (2019). Neurotransmitter imbalance in the brain and Alzheimer’s disease pathology. *Journal of Alzheimer’s Disease*.

[87] Coyle J., Price D., DeLong M. (1983). Alzheimer’s disease: a disorder of cortical cholinergic innervation. *Science*.

[88] Whitehouse P., Price D., Struble R., Clark A., Coyle J., Delon M. (1982). Alzheimer’s disease and senile dementia: loss of neurons in the basal forebrain. *Science*.

[89] Wang X., Wang W., Li L., Perry G., Lee H.-g., Zhu X. (2014). Oxidative stress and mitochondrial dysfunction in Alzheimer’s disease. *Biochimica et Biophysica Acta (BBA)—Molecular Basis of Disease*.

[90] Owen J. B., Sultana R., Aluise C. D. (2010). Oxidative modification to LDL receptor-related protein 1 in hippocampus from subjects with Alzheimer disease: implications for A*β* accumulation in AD brain. *Free Radical Biology and Medicine*.

[91] Love S., Miners J. S. (2016). Cerebrovascular disease in ageing and Alzheimer’s disease. *Acta Neuropathologica*.

[92] Bennett R. E., Robbins A. B., Hu M. (2018). Tau induces blood vessel abnormalities and angiogenesis-related gene expression in P301L transgenic mice and human Alzheimer’s disease. *Proceedings of the National Academy of Sciences*.

[93] Fanning S. (2018). Lipidomic analysis of alpha-synuclein neurotoxicity identifies stearoyl CoA desaturase as a target for Parkinson treatment. *Molecular Cell*.

[94] Zijlmans J. C. M., Daniel S. E., Hughes A. J., Révész T., Lees A. J. (2004). Clinicopathological investigation of vascular parkinsonism, including clinical criteria for diagnosis. *Movement Disorders*.

[95] Benedictus M. R., Binnewijzend M. A. A., Kuijer J. P. A. (2014). Brain volume and white matter hyperintensities as determinants of cerebral blood flow in Alzheimer’s disease. *Neurobiology of Aging*.

[96] Kouhestani S., Jafari A., Babaei P. (2018). Kaempferol attenuates cognitive deficit via regulating oxidative stress and neuroinflammation in an ovariectomized rat model of sporadic dementia. *Neural Regeneration Research*.

[97] Balez R. (2016). Neuroprotective effects of apigenin against inflammation, neuronal excitability and apoptosis in an induced pluripotent stem cell model of Alzheimer’s disease. *Scientific Reports*.

[98] Burg V. K., Grimm H. S., Rothhaar T. L. (2013). Plant sterols the better cholesterol in Alzheimer’s disease? A mechanistical study. *Journal of Neuroscience*.

[99] Kwon Y. (2017). Luteolin as a potential preventive and therapeutic candidate for Alzheimer’s disease. *Experimental Gerontology*.

[100] Jiancheng H., Wenwu W. (2010). Effect of Tianma Gouteng Yin on apoptosis of dopaminergic neurons in Parkinson’s disease model rats. *Journal of Traditional Chinese Medicine*.

[101] Liu L. F. (2015). Tianma Gouteng Yin, a traditional Chinese medicine decoction, exerts neuroprotective effects in animal and cellular models of Parkinson’s disease. *Scientific Reports*.

[102] Yuchi G., Zhaochen J., Jingbo L. (2018). Prescription regularity of traditional Chinese medicine treatment for Parkinson disease: based on complex network analysis. *Journal of Traditional Chinese Medicine*.

[103] Munoz L., Ammit A. J. (2010). Targeting p38 MAPK pathway for the treatment of Alzheimer’s disease. *Neuropharmacology*.

[104] Gao J. (2016). HDAC3 but not HDAC2 mediates visual experience-dependent radial glia proliferation in the developing Xenopus tectum. *Frontiers in Cellular Neuroscience*.

[105] Kong Y., Liang X., Liu L. (2015). High throughput sequencing identifies microRNAs mediating alpha-synuclein toxicity by targeting neuroactive-ligand receptor interaction pathway in early stage of drosophila Parkinson’s disease model. *PLoS One*.

[106] Zhu Y., Duan X., Cheng X. (2016). Kai-Xin-San, a standardized traditional Chinese medicine formula, up-regulates the expressions of synaptic proteins on hippocampus of chronic mild stress induced depressive rats and primary cultured rat hippocampal neuron. *Journal of Ethnopharmacology*.

[107] Adkins D. E. (2012). SNP-based analysis of neuroactive ligand-receptor interaction pathways implicates PGE2 as a novel mediator of antipsychotic treatment response: data from the CATIE study. *Schizophrenia Research*.

[108] Li X. J., Kim K.-W., Oh H., Liu X.-Q., Kim Y.-C. (2019). Chemical constituents and an antineuroinflammatory lignan, savinin from the roots of acanthopanax henryi. *Evidence-Based Complementary and Alternative Medicine*.

[109] Liu A., Zhao X., Li H. (2014). 5-Hydroxymethylfurfural, an antioxidant agent from Alpinia oxyphylla miq. improves cognitive impairment in A*β*1-42 mouse model of Alzheimer’s disease. *International Immunopharmacology*.

[110] Booij B. B., Lindahl T., Wetterberg P. (2011). A gene expression pattern in blood for the early detection of Alzheimer’s disease. *Journal of Alzheimer’s Disease*.

[111] Fehlbaum-Beurdeley P., Jarrige-Le Prado A. C., Pallares D. (2010). Toward an Alzheimer’s disease diagnosis via high-resolution blood gene expression. *Alzheimer’s & Dementia*.

[112] Spires-Jones T. L., Hyman B. T. (2014). The intersection of amyloid beta and tau at synapses in Alzheimer’s disease. *Neuron*.

[113] Ballard C., Gauthier S., Corbett A., Brayne C., Aarsland D., Jones E. (2011). Alzheimer’s disease. *The Lancet*.

[114] Dejanovic B., Huntley M. A., Mazière A. D. (2018). Changes in the synaptic proteome in tauopathy and rescue of tau-induced synapse loss by C1q antibodies. *Neuron*.

[115] Palotás A., Reis H. J., Bogáts G. (2010). Coronary artery bypass surgery provokes Alzheimer’s disease-like changes in the cerebrospinal fluid. *Journal of Alzheimer’s Disease*.

[116] Lee T. A., Wolozin B., Weiss K. B., Bednar M. M. (2005). Assessment of the emergence of Alzheimer’s disease following coronary artery bypass graft surgery or percutaneous transluminal coronary angioplasty. *Journal of Alzheimer’s Disease*.

[117] Horenstein R. B., Mitchell B. D., Post W. S. (2013). The ABCG8 G574R variant, serum plant sterol levels, and cardiovascular disease risk in the old order amish. *Arteriosclerosis, Thrombosis, and Vascular Biology*.

[118] Jeong S. I., Kim K. J., Choi M. K. (2004). *α*-Spinasterol isolated from the root of Phytolacca americana and its pharmacological property on diabetic nephropathy. *Planta Medica*.

[119] Zhu L., Wang P., Yuan W., Zhu G. (2018). Kaempferol inhibited bovine herpesvirus 1 replication and LPS-induced inflammatory response. *Acta Virologica*.

[120] Deliorman D. (2000). Studies on the vascular effects of the fractions and phenolic compounds isolated from Viscum album ssp. album. *Journal for Ethnopharmacology*.

[121] Nagatsu T., Mogi M., Ichinose H., Togari A. (2000). Changes in cytokines and neurotrophins in Parkinson’s disease. *Advances in Research on Neurodegeneration*.

[122] Peter I., Dubinsky M., Bressman S. (2018). Anti-tumor necrosis factor therapy and incidence of Parkinson disease among patients with inflammatory bowel disease. *JAMA Neurology*.

[123] Steinert A., Linas I., Kaya B. (2017). The stimulation of macrophages with TLR ligands supports increased IL-19 expression in inflammatory bowel disease patients and in colitis models. *The Journal of Immunology*.

[124] Kam T. I. (2018). Poly (ADP-ribose) drives pathologic alpha-synuclein neurodegeneration in Parkinson’s disease. *Science*.

[125] Cookson M. R. (2010). The role of leucine-rich repeat kinase 2 (LRRK2) in Parkinson’s disease. *Nature Reviews‘ Neuroscience*.

[126] Healy D. G., Falchi M., O’Sullivan S. S. (2008). Phenotype, genotype, and worldwide genetic penetrance of LRRK2-associated Parkinson’s disease: a case-control study. *The Lancet Neurology*.

[127] Subramaniam S. R., Chesselet M.-F. (2013). Mitochondrial dysfunction and oxidative stress in Parkinson’s disease. *Progress in Neurobiology*.

[128] Jiang T., Sun Q., Chen S. (2016). Oxidative stress: a major pathogenesis and potential therapeutic target of antioxidative agents in Parkinson’s disease and Alzheimer’s disease. *Progress in Neurobiology*.

[129] Korczyn A. D. (2015). Vascular parkinsonism-characteristics, pathogenesis and treatment. *Nature Reviews Neurology*.

