# Supplementary material for: Enhancing and Complementary Mechanisms of Synergistic Action of Acori Tatarinowii Rhizoma and Codonopsis Radix for Alzheimer's Disease Based on Systems Pharmacology
Source: Evid Based Complement Alternat Med. 2020 Jun 25;2020:6317230. doi: 10.1155/2020/6317230 (PMC7334796; doi:10.1155/2020/6317230)
Supplement: Supplementary Materials — Figure S1. The number of targets of ATR (red), CR (blue), and Alzheimer's disease (green) obtained from databases. Figure S2. CS and accumulative CS of the active ingredients in ATR or CR. Table S1. Detailed information on the ingredients in ATR and CR. Table S2. The value of active ingredients in ATR and CR. [file 6317230.f1.zip › 6317230.f1/S1 Table.docx]

**Table S1** The detail information of the ingredients in ShiChangPu and DangShen

| **ID** | **Molecule Name** | **MW** | **AlogP** | **nHdon** | **nHacc** | **OB (%)** | **DL** |
| --- | --- | --- | --- | --- | --- | --- | --- |
| DS1 | poriferasta-7,22E-dien-3beta-ol | 412.77 | 7.64 | 1 | 1 | 42.98 | 0.76 |
| DS2 | 2-methoxyfuranodiene | 246.38 | 3.94 | 0 | 2 | 53.58 | 0.13 |
| DS3 | (-)-alpha-Pinene | 136.26 | 2.87 | 0 | 0 | 46.25 | 0.05 |
| DS4 | 6-methylolpyridin-3-ol | 125.14 | 0.24 | 2 | 3 | 47.53 | 0.02 |
| DS5 | EIC | 280.5 | 6.39 | 1 | 2 | 41.9 | 0.14 |
| DS6 | Azelex | 188.25 | 1.87 | 2 | 4 | 16.9 | 0.04 |
| DS7 | Methyl myristate | 242.45 | 5.71 | 0 | 2 | 19.68 | 0.08 |
| DS8 | myristic acid | 228.42 | 5.46 | 1 | 2 | 21.18 | 0.07 |
| DS9 | Oktadekan | 254.56 | 8.58 | 0 | 0 | 9.81 | 0.09 |
| DS10 | PENTADECYLIC ACID | 242.45 | 5.91 | 1 | 2 | 20.18 | 0.08 |
| DS11 | TWT | 310.68 | 10.41 | 0 | 0 | 8.37 | 0.18 |
| DS12 | UPL | 268.59 | 9.04 | 0 | 0 | 8.52 | 0.11 |
| DS13 | Pentadecene | 210.45 | 6.82 | 0 | 0 | 17.72 | 0.05 |
| DS14 | METHYL LINOLEATE | 294.53 | 6.64 | 0 | 2 | 41.93 | 0.17 |
| DS15 | Dodecanal | 184.36 | 4.59 | 0 | 1 | 21.52 | 0.03 |
| DS16 | (+/-)-Isoborneol | 154.28 | 1.98 | 1 | 1 | 86.98 | 0.05 |
| DS17 | Methyl stearate | 298.57 | 7.53 | 0 | 2 | 16.8 | 0.16 |
| DS18 | METHYL PENTADECANOATE | 256.48 | 6.16 | 0 | 2 | 18.82 | 0.1 |
| DS19 | SRT | 150.1 | -1.5 | 4 | 6 | 45.27 | 0.02 |
| DS20 | hexanoic acid | 116.18 | 1.81 | 1 | 2 | 73.08 | 0.01 |
| DS21 | Perlolyrine | 264.3 | 3.2 | 2 | 3 | 65.95 | 0.27 |
| DS22 | 20-Hexadecanoylingenol | 586.94 | 7.38 | 3 | 6 | 28.2 | 0.68 |
| DS23 | beta-Curcumene | 204.39 | 5.33 | 0 | 0 | 4.48 | 0.06 |
| DS24 | 3691-11-0 | 204.39 | 4.99 | 0 | 0 | 23.66 | 0.07 |
| DS25 | capsaicin | 305.46 | 3.89 | 2 | 4 | 10.31 | 0.2 |
| DS26 | Myristicin | 192.23 | 2.59 | 0 | 3 | 17.99 | 0.07 |
| DS27 | alpha-Curcumene | 202.37 | 5.34 | 0 | 0 | 4.68 | 0.06 |
| DS28 | Diop | 390.62 | 7.44 | 0 | 4 | 43.59 | 0.39 |
| DS29 | BuOH | 74.14 | 0.97 | 1 | 1 | 22.02 | 0 |
| DS30 | caprylic acid | 144.24 | 2.72 | 1 | 2 | 16.4 | 0.02 |
| DS31 | stigmasterol-β-glucoside | 574.93 | 5.89 | 4 | 6 | 2.4 | 0.63 |
| DS32 | ZINC03978781 | 412.77 | 7.64 | 1 | 1 | 43.83 | 0.76 |
| DS33 | lauric acid | 200.36 | 4.54 | 1 | 2 | 23.59 | 0.04 |
| DS34 | nonanoic acid | 158.27 | 3.17 | 1 | 2 | 40.51 | 0.02 |
| DS35 | Syringaldehyde | 182.19 | 1.29 | 1 | 4 | 67.06 | 0.05 |
| DS36 | Hentriacontan | 436.95 | 14.51 | 0 | 0 | 8.07 | 0.51 |
| DS37 | Stigmasterol | 412.77 | 7.64 | 1 | 1 | 43.83 | 0.76 |
| DS38 | Syrigin | 372.41 | -0.51 | 5 | 9 | 14.64 | 0.32 |
| DS39 | D-Friedoolean-14-en-3-one | 424.78 | 7.26 | 0 | 1 | 12.9 | 0.77 |
| DS40 | Nonanol | 144.29 | 3.25 | 1 | 1 | 33.19 | 0.01 |
| DS41 | syringaresinol | 418.48 | 2.1 | 2 | 8 | 3.29 | 0.72 |
| DS42 | Shekanin | 462.44 | 0.14 | 6 | 11 | 25.1 | 0.79 |
| DS43 | tectorigenin | 300.28 | 2.05 | 3 | 6 | 28.41 | 0.27 |
| DS44 | 5-Methoxymethyl furfural | 140.15 | 1.08 | 0 | 3 | 28.23 | 0.02 |
| DS45 | 7-Methoxy-2-methyl isoflavone | 266.31 | 3.36 | 0 | 3 | 42.56 | 0.2 |
| DS46 | choline | 104.2 | -1.57 | 1 | 1 | 0.47 | 0.01 |
| DS47 | nicotinic acid | 123.12 | 0.28 | 1 | 3 | 47.65 | 0.02 |
| DS48 | Spinasterol | 412.77 | 7.64 | 1 | 1 | 42.98 | 0.76 |
| DS49 | atractylenolideII | 232.35 | 3.57 | 0 | 2 | 47.5 | 0.15 |
| DS50 | Chrysanthemaxanthin | 584.96 | 8.24 | 2 | 3 | 38.72 | 0.58 |
| DS51 | 12-O-Nicotinoylisolineolone | 469.63 | 1.32 | 3 | 7 | 20.7 | 0.83 |
| DS52 | atractylenolide iii | 248.35 | 2.93 | 1 | 3 | 68.11 | 0.17 |
| DS53 | Methyl naphthalene | 142.21 | 3.23 | 0 | 0 | 39.01 | 0.04 |
| DS54 | Encecalin | 232.3 | 2.34 | 0 | 3 | 21.36 | 0.11 |
| DS55 | (2R,3R,4S,5S,6R)-2-[[(3S,5S,9R,10S,13R,14R,17R)-17-[(E,2R,5S)-5-ethyl-6-methylhept-3-en-2-yl]-10,13-dimethyl-2,3,4,5,6,9,11,12,14,15,16,17-dodecahydro-1H-cyclopenta[a]phenanthren-3-yl]oxy]-6-(hydroxymethyl)oxane-3,4,5-triol | 574.93 | 5.89 | 4 | 6 | 21.2 | 0.63 |
| DS56 | heptanoic acid | 130.21 | 2.26 | 1 | 2 | 13.38 | 0.01 |
| DS57 | Friedelin | 426.8 | 7.3 | 0 | 1 | 29.16 | 0.76 |
| DS58 | n-Heptadecanol | 256.53 | 6.9 | 1 | 1 | 12.97 | 0.09 |
| DS59 | 7-(beta-Xylosyl)cephalomannine | 962.15 | 1.97 | 6 | 18 | 27.33 | 0.17 |
| DS60 | 7alpha-L-Rhamnosyl-6-methoxylutcolin | 462.44 | 1.03 | 6 | 11 | 15.03 | 0.79 |
| DS61 | Frutinone A | 264.24 | 2.7 | 0 | 4 | 65.9 | 0.34 |
| DS62 | FOA | 112.09 | 0.83 | 1 | 3 | 35.66 | 0.02 |
| DS63 | luteolin | 286.25 | 2.07 | 4 | 6 | 36.16 | 0.25 |
| DS64 | (14S)-14-methylpalmitic acid | 270.51 | 6.62 | 1 | 2 | 23.12 | 0.11 |
| DS65 | darutoside | 574.93 | 5.89 | 4 | 6 | 21.32 | 0.63 |
| DS66 | Friedoolean-14-en-3-yl acetate | 468.84 | 7.68 | 0 | 2 | 9.59 | 0.74 |
| DS67 | Taraxerol | 426.8 | 7.3 | 1 | 1 | 38.4 | 0.77 |
| DS68 | 1-hexanol | 102.2 | 1.88 | 1 | 1 | 22.04 | 0.01 |
| DS69 | stigmast-7-enol | 414.79 | 8.08 | 1 | 1 | 37.42 | 0.75 |
| DS70 | Norharman | 168.21 | 2.17 | 1 | 1 | 18.88 | 0.08 |
| DS71 | palmitic acid | 256.48 | 6.37 | 1 | 2 | 19.3 | 0.1 |
| DS72 | NSC405997 | 342.68 | 7.96 | 2 | 2 | 12.59 | 0.26 |
| DS73 | 3-beta-Hydroxymethyllenetanshiquinone | 294.32 | 3.16 | 1 | 4 | 32.16 | 0.41 |
| DS74 | Nonadienal | 138.23 | 2.75 | 0 | 1 | 19.03 | 0.02 |
| DS75 | HMF | 126.12 | 0.67 | 1 | 3 | 45.07 | 0.02 |
| DS76 | methyl icosa-11,14-dienoate | 322.59 | 7.55 | 0 | 2 | 39.67 | 0.23 |
| DS77 | apigenin | 270.25 | 2.33 | 3 | 5 | 23.06 | 0.21 |
| DS78 | Ricinin | 164.18 | -0.78 | 0 | 4 | 26.26 | 0.04 |
| DS79 | BUA | 88.12 | 0.89 | 1 | 2 | 21.62 | 0 |
| DS80 | EA-fructofuranoside | 208.24 | -1.81 | 4 | 6 | 47.33 | 0.06 |
| DS81 | (1R)-2,3,4,9-tetrahydro-1H-pyrido[3,4-b]indol-2-ium-1-carboxylate | 216.26 | 1.73 | 3 | 3 | 52.9 | 0.13 |
| DS82 | 13-Methyl pentadecanoic acid | 256.48 | 6.16 | 1 | 2 | 24.14 | 0.1 |
| DS83 | Galuteolin | 448.41 | 0.16 | 7 | 11 | 2.7 | 0.79 |
| DS84 | o-(o-Methoxyphenoxy)phenol | 216.25 | 3.11 | 1 | 3 | 50.75 | 0.09 |
| DS85 | tangshenoside I | 678.71 | -2.37 | 9 | 18 | 4.93 | 0.47 |
| DS86 | tangshenoside I_qt | 354.39 | 1.29 | 3 | 8 | 23.58 | 0.3 |
| DS87 | tangshenoside III | 726.8 | -0.27 | 9 | 17 | 8.75 | 0.46 |
| DS88 | tangshenoside III_qt | 402.48 | 3.54 | 3 | 7 | 4.98 | 0.45 |
| DS89 | tangshenoside IV | 1,033.10 | -2.13 | 13 | 26 | 3.01 | 0.15 |
| DS90 | tangshenoside IV_qt | 546.62 | 3.43 | 4 | 11 | 4.28 | 0.67 |
| DS91 | T-BUTYLBENZENE | 134.24 | 3.23 | 0 | 0 | 53.06 | 0.02 |
| DS92 | 2,6-NONADIENOL | 140.25 | 2.5 | 1 | 1 | 23.77 | 0.02 |
| DS93 | 3-METHYLCARBAZOLE | 181.25 | 3.81 | 1 | 0 | 20.14 | 0.09 |
| DS94 | L-Sulforaphane | 177.32 | 1.16 | 0 | 2 | 56.97 | 0.02 |
| DS95 | 4-Phenylbicyclo[2,2,2]octan-1-ol | 202.32 | 2.89 | 1 | 1 | 25.07 | 0.09 |
| DS96 | 5-Mpe-bis(hobz)phenol | 438.55 | 6.95 | 3 | 4 | 11.33 | 0.66 |
| DS97 | 5alpha-Stigmastan-3,6-dione | 428.77 | 6.66 | 0 | 2 | 33.12 | 0.79 |
| DS98 | 6,6'-Dimethoxygossypol | 550.7 | 7.39 | 4 | 8 | 8.93 | 0.86 |
| DS99 | 7-(beta-Xylosyl)cephalomannine_qt | 830.02 | 3.21 | 4 | 14 | 38.33 | 0.29 |
| DS100 | Butylcyclohexane | 140.3 | 4.36 | 0 | 0 | 35.68 | 0.02 |
| DS101 | Codonopsine | 267.36 | 1.07 | 2 | 5 | 45.83 | 0.13 |
| DS102 | Coelogin | 300.33 | 3.12 | 2 | 5 | 21.68 | 0.42 |
| DS103 | Daturilin | 436.64 | 4.34 | 0 | 4 | 50.37 | 0.77 |
| DS104 | Ethyl-p-digallate | 350.3 | 2.13 | 5 | 9 | 2.27 | 0.31 |
| DS105 | fritillaziebinol | 324.98 | 4.93 | 1 | 1 | 15.01 | 0.34 |
| DS106 | glycitein | 284.28 | 2.32 | 2 | 5 | 50.48 | 0.24 |
| DS107 | Henicosanoic acid | 326.63 | 8.65 | 1 | 2 | 16.14 | 0.23 |
| DS108 | BHG | 264.36 | 0.14 | 4 | 6 | 15.6 | 0.11 |
| DS109 | Hexyl-beta-D-glucopyranosyl-(1-2)-beta-D-glucopyranoside | 426.52 | -1.61 | 7 | 11 | 6.51 | 0.44 |
| DS110 | (2S)-2-ammonio-4-[(R)-methylsulfinyl]butyrate | 165.24 | -1.38 | 3 | 4 | 83.52 | 0.02 |
| DS111 | 1-Peroxyferolide | 338.39 | 1.44 | 1 | 7 | 17.38 | 0.35 |
| DS112 | Spinoside A | 716.95 | 2.91 | 5 | 12 | 39.97 | 0.4 |
| DS113 | (8S,9S,10R,13R,14S,17R)-17-[(E,2R,5S)-5-ethyl-6-methylhept-3-en-2-yl]-10,13-dimethyl-1,2,4,7,8,9,11,12,14,15,16,17-dodecahydrocyclopenta[a]phenanthren-3-one | 410.75 | 7.31 | 0 | 1 | 45.4 | 0.76 |
| DS114 | Stigmasteryl ferulate | 588.95 | 9.87 | 1 | 4 | 24.53 | 0.55 |
| DS115 | Tangshenoside II | 372.41 | -0.33 | 5 | 9 | 19.5 | 0.32 |
| DS116 | Tangshenoside II_qt | 210.25 | 1.57 | 2 | 4 | 51.72 | 0.06 |
| DS117 | 11-Hydroxyrankinidine | 356.46 | 1.04 | 2 | 6 | 40 | 0.66 |
| DS118 | alpha-Stigmasta-7,22-dien-3-one | 410.75 | 7.31 | 0 | 1 | 11.42 | 0.76 |
| DS119 | Codopiloic acid | 127.11 | -1.06 | 1 | 4 | 57.5 | 0.02 |
| DS120 | delta22-Stigmasterol | 414.79 | 7.89 | 1 | 1 | 7.04 | 0.76 |
| DS121 | delta7-Stigmastenone-3 | 412.77 | 7.76 | 0 | 1 | 9.69 | 0.76 |
| DS122 | delta7-stigmastenol-belta-D-glucopyranoside | 578.97 | 6.59 | 4 | 6 | 19.58 | 0.62 |
| DS123 | ethyl-β-D-fructofuranoside | 320.48 | 1.22 | 0 | 6 | 33.84 | 0.15 |
| DS124 | stearic acid | 284.54 | 7.28 | 1 | 2 | 17.83 | 0.14 |
| DS125 | Heptadekan | 240.53 | 8.13 | 0 | 0 | 8.64 | 0.07 |
| DS126 | Henicosane | 296.65 | 9.95 | 0 | 0 | 8.41 | 0.15 |
| DS127 | methyl palmitate | 270.51 | 6.62 | 0 | 2 | 18.09 | 0.12 |
| DS128 | Dodekan | 170.38 | 5.85 | 0 | 0 | 17.74 | 0.02 |
| DS129 | (+)-alpha-Curcumene | 202.37 | 5.34 | 0 | 0 | 26.56 | 0.06 |
| DS130 | Furanodiene | 216.35 | 4.63 | 0 | 1 | 45.11 | 0.1 |
| DS131 | luteolin-7-o-glucoside | 448.41 | 0.16 | 7 | 11 | 7.29 | 0.78 |
| DS132 | (+)-beta-Pinene | 136.26 | 2.93 | 0 | 0 | 44.77 | 0.05 |
| DS133 | delta 7-stigmastenol | 416.81 | 8.33 | 1 | 1 | 25.32 | 0.75 |
| DS134 | 2-(3,4-dihydroxyphenyl)-5,7-dihydroxy-3-[(2R,3R,4S,5S)-3,4,5-trihydroxytetrahydropyran-2-yl]oxy-chromone | 434.38 | -0.08 | 7 | 11 | 4.05 | 0.7 |
| SCP1 | Rhoifolin | 578.57 | -0.43 | 8 | 14 | 6.68 | 0.77 |
| SCP2 | protocatechuic acid | 154.13 | 0.9 | 3 | 4 | 25.37 | 0.04 |
| SCP3 | vanillic acid | 168.16 | 1.15 | 2 | 4 | 35.47 | 0.04 |
| SCP4 | Cymol | 134.24 | 3.51 | 0 | 0 | 27.2 | 0.02 |
| SCP5 | alpha-Cubebene | 204.39 | 4.17 | 0 | 0 | 16.73 | 0.11 |
| SCP6 | (-)-Alloaromadendrene | 204.39 | 4.22 | 0 | 0 | 54.04 | 0.1 |
| SCP7 | ZINC02040970 | 222.41 | 4.56 | 1 | 1 | 40.43 | 0.06 |
| SCP8 | CAM | 152.26 | 1.94 | 0 | 1 | 67.17 | 0.05 |
| SCP9 | WLN: Q1R | 152.26 | 1.23 | 1 | 1 | 58.68 | 0.01 |
| SCP10 | myristic acid | 152.26 | 5.46 | 1 | 2 | 21.18 | 0.07 |
| SCP11 | ZINC01081275 | 152.26 | 3.02 | 0 | 1 | 43.92 | 0.05 |
| SCP12 | calarene | 152.26 | 4.12 | 0 | 0 | 52.16 | 0.11 |
| SCP13 | α-cubebol | 152.26 | 3.28 | 1 | 1 | 64.81 | 0.09 |
| SCP14 | ZINC01609418 | 152.26 | 4.31 | 1 | 1 | 21.62 | 0.07 |
| SCP15 | p-MCA | 152.26 | 1.89 | 1 | 3 | 31 | 0.05 |
| SCP16 | Guaiol | 152.26 | 3.91 | 1 | 1 | 38.77 | 0.09 |
| SCP17 | D-Camphene | 152.26 | 2.93 | 0 | 0 | 34.98 | 0.04 |
| SCP18 | (Z)-caryophyllene | 152.26 | 4.75 | 0 | 0 | 30.29 | 0.09 |
| SCP19 | Marmesin | 152.26 | 2.03 | 1 | 4 | 50.28 | 0.18 |
| SCP20 | Majudin | 152.26 | 2.19 | 0 | 4 | 42.21 | 0.13 |
| SCP21 | Myrcene | 152.26 | 3.69 | 0 | 0 | 24.96 | 0.02 |
| SCP22 | (R)-linalool | 152.26 | 2.74 | 1 | 1 | 39.8 | 0.02 |
| SCP23 | (-)-Caryophyllene oxide | 152.26 | 3.52 | 0 | 1 | 32.67 | 0.13 |
| SCP24 | Isocembrol | 152.26 | 6.01 | 1 | 1 | 15.06 | 0.17 |
| SCP25 | thymol | 152.26 | 3.24 | 1 | 1 | 41.47 | 0.03 |
| SCP26 | Methyleugenol | 152.26 | 2.81 | 0 | 2 | 73.36 | 0.04 |
| SCP27 | DEP | 152.26 | 2.24 | 0 | 4 | 52.19 | 0.07 |
| SCP28 | beta-asarone | 152.26 | 2.74 | 0 | 3 | 35.61 | 0.06 |
| SCP29 | caffeic acid | 152.26 | 1.37 | 3 | 4 | 25.76 | 0.05 |
| SCP30 | beta-Gurjunene | 152.26 | 4.22 | 0 | 0 | 51.36 | 0.1 |
| SCP31 | Terragon | 152.26 | 2.82 | 0 | 1 | 36.59 | 0.03 |
| SCP32 | alpha-humulene | 152.26 | 5.04 | 0 | 0 | 22.98 | 0.06 |
| SCP33 | (-)-beta-Phellandrene | 152.26 | 3.31 | 0 | 0 | 40.44 | 0.02 |
| SCP34 | ISOELEMICIN | 152.26 | 2.74 | 0 | 3 | 46.72 | 0.06 |
| SCP35 | beta-Cubebene | 152.26 | 4.22 | 0 | 0 | 32.81 | 0.11 |
| SCP36 | (1S,5S)-1-isopropyl-4-methylenebicyclo[3.1.0]hexane | 152.26 | 2.93 | 0 | 0 | 46.21 | 0.04 |
| SCP37 | Elemicin | 152.26 | 2.79 | 0 | 3 | 21.94 | 0.06 |
| SCP38 | nicotiflorin | 152.26 | -1.18 | 9 | 15 | 3.64 | 0.73 |
| SCP39 | beta-Humulene | 152.26 | 5.09 | 0 | 0 | 26.87 | 0.06 |
| SCP40 | 2'-O-Methylisoliquiritigenin | 152.26 | 3.15 | 2 | 4 | 75.86 | 0.17 |
| SCP41 | MTL | 152.26 | -2.94 | 6 | 6 | 17.73 | 0.03 |
| SCP42 | BZM | 152.26 | 3.27 | 0 | 2 | 18.64 | 0.09 |
| SCP43 | [(1S)-endo]-(-)-Borneol | 152.26 | 1.98 | 1 | 1 | 83.54 | 0.05 |
| SCP44 | alpha cadinene | 152.26 | 4.75 | 0 | 0 | 18.73 | 0.08 |
| SCP45 | 2,5-Dimethoxybenzoquinone | 152.26 | -0.59 | 0 | 4 | 8.72 | 0.04 |
| SCP46 | Veraguensin | 152.26 | 4.31 | 0 | 5 | 25.53 | 0.39 |
| SCP47 | beta-Selinene | 152.26 | 4.81 | 0 | 0 | 24.39 | 0.08 |
| SCP48 | Isohomogenol | 152.26 | 2.75 | 0 | 2 | 32.61 | 0.04 |
| SCP49 | CADINENE | 152.26 | 4.75 | 0 | 0 | 17.12 | 0.08 |
| SCP50 | (+)-Ledene | 152.26 | 4.36 | 0 | 0 | 51.84 | 0.1 |
| SCP51 | (E)-2-propenoic acid,3-(3-hydroxy-2,6,6-trimethyl-1-cyclohexen-1-yl),methyl ester | 152.26 | 2.38 | 1 | 3 | 30.7 | 0.08 |
| SCP52 | (1R)-Camphor-10-sulfonic acid | 152.26 | 0.42 | 1 | 4 | 42.61 | 0.09 |
| SCP53 | (+)-alpha-Longipinene | 152.26 | 4.12 | 0 | 0 | 57.47 | 0.12 |
| SCP54 | 8-Isopentenyl-kaempferol | 152.26 | 3.63 | 4 | 6 | 38.04 | 0.39 |
| SCP55 | Aminacrin | 152.26 | 2.61 | 2 | 2 | 35 | 0.12 |
| SCP56 | Acoramone | 152.26 | 1.41 | 0 | 4 | 63.43 | 0.07 |
| SCP57 | aristolene | 152.26 | 4.12 | 0 | 0 | 52.2 | 0.11 |
| SCP58 | Aristolone | 152.26 | 3.19 | 0 | 1 | 45.31 | 0.13 |
| SCP59 | Azaron | 152.26 | 2.74 | 0 | 3 | 38.39 | 0.06 |
| SCP60 | Asatone | 152.26 | 0.91 | 0 | 8 | 12.53 | 0.52 |
| SCP61 | gamma-Asarone | 152.26 | 2.79 | 0 | 3 | 22.76 | 0.06 |
| SCP62 | WLN: G1R | 152.26 | 2.43 | 0 | 0 | 73.8 | 0.01 |
| SCP63 | Bisasarcin | 152.26 | 4.8 | 0 | 6 | 18.55 | 0.5 |
| SCP64 | Cyclite | 152.26 | 2.58 | 0 | 0 | 9.13 | 0.01 |
| SCP65 | Calamendiol | 152.26 | 2.51 | 2 | 2 | 61.13 | 0.11 |
| SCP66 | Gazarin | 152.26 | 1.54 | 0 | 4 | 74.63 | 0.06 |
| SCP67 | Methyl isoeugenol | 152.26 | 2.75 | 0 | 2 | 74.21 | 0.04 |
| SCP68 | δ-cadinene | 152.26 | 4.94 | 0 | 0 | 20.29 | 0.08 |
| SCP69 | (-)-Valencene | 152.26 | 4.75 | 0 | 0 | 37.63 | 0.08 |
| SCP70 | Asaronic acid | 152.26 | 1.39 | 1 | 5 | 61.92 | 0.07 |
| SCP71 | gramenone | 152.26 | 4.02 | 0 | 1 | 16.36 | 0.09 |
| SCP72 | lupeol | 152.26 | 7.4 | 1 | 1 | 12.12 | 0.78 |
| SCP73 | Isocalamendiol | 152.26 | 2.69 | 2 | 2 | 57.63 | 0.11 |
| SCP74 | isopimpinellin | 152.26 | 2.17 | 0 | 5 | 25.93 | 0.17 |
| SCP75 | Isoschaftoside | 152.26 | -1.94 | 10 | 14 | 17.38 | 0.83 |
| SCP76 | Isoshyobunone | 152.26 | 4.19 | 0 | 1 | 23.48 | 0.07 |
| SCP77 | (E)-3-(2,4-dimethylphenyl)acrylic acid | 152.26 | 2.88 | 1 | 2 | 37.86 | 0.05 |
| SCP78 | longicyclene | 152.26 | 3.55 | 0 | 0 | 46.07 | 0.15 |
| SCP79 | Murolan-3,9(11)-diene-10-peroxy | 152.26 | 3.99 | 1 | 2 | 36.72 | 0.11 |
| SCP80 | Diethylbenzylamine | 152.26 | 2.6 | 0 | 1 | 43.05 | 0.03 |
| SCP81 | Patchoulene | 152.26 | 4.32 | 0 | 0 | 49.06 | 0.11 |
| SCP82 | 3,5,8-trihydroxy-2-(4-hydroxyphenyl)-7-[(2S,3R,4R,5R,6S)-3,4,5-trihydroxy-6-methyl-tetrahydropyran-2-yl]oxy-chromone | 152.26 | 0.49 | 7 | 11 | 21.69 | 0.75 |
| SCP83 | Epishyobunone | 152.26 | 3.84 | 0 | 1 | 42.37 | 0.07 |
| SCP84 | spathulenol | 152.26 | 3.01 | 1 | 1 | 81.61 | 0.12 |
| SCP85 | ZINC01849758 | 152.26 | 4.31 | 1 | 1 | 16.77 | 0.07 |
| SCP86 | calacorene | 152.26 | 4.79 | 0 | 0 | 16.2 | 0.08 |
| SCP87 | α-gurjunene | 152.26 | 4.36 | 0 | 0 | 52.57 | 0.1 |
| SCP88 | α-Panasinsene | 152.26 | 4.08 | 0 | 0 | 56.77 | 0.12 |
| SCP89 | (1R,3aS,4R,6aS)-1,4-bis(3,4-dimethoxyphenyl)-1,3,3a,4,6,6a-hexahydrofuro[4,3-c]furan | 152.26 | 2.64 | 0 | 6 | 52.35 | 0.62 |
| SCP90 | Bisasaricin | 152.26 | 4.8 | 0 | 6 | 28.94 | 0.5 |
| SCP91 | Cycloartenol | 152.26 | 7.55 | 1 | 1 | 38.69 | 0.78 |
| SCP92 | Anizol | 152.26 | 1.81 | 0 | 1 | 19.88 | 0.01 |
| SCP93 | kaempferol | 152.26 | 1.77 | 4 | 6 | 41.88 | 0.24 |
| SCP94 | emodin | 152.26 | 2.49 | 3 | 5 | 24.4 | 0.24 |
| SCP95 | (-)-trans-Carveol | 152.26 | 2.4 | 1 | 1 | 45.28 | 0.03 |
| SCP96 | Astragalin | 152.26 | -0.32 | 7 | 11 | 14.03 | 0.74 |
| SCP97 | (+)-Terpinen-4-ol | 152.26 | 2.55 | 1 | 1 | 81.41 | 0.03 |
| SCP98 | (-)-alpha-cedrene | 152.26 | 4.12 | 0 | 0 | 55.56 | 0.1 |
| SCP99 | palmitic acid | 152.26 | 6.37 | 1 | 2 | 19.3 | 0.1 |
| SCP100 | WLN: VHR | 152.26 | 1.59 | 0 | 1 | 32.63 | 0.01 |
| SCP101 | HMF | 152.26 | 0.67 | 1 | 3 | 45.07 | 0.02 |
| SCP102 | p-coumaric acid | 152.26 | 1.64 | 2 | 3 | 43.29 | 0.04 |
| SCP103 | apigenin | 152.26 | 2.33 | 3 | 5 | 23.06 | 0.21 |
| SCP104 | Azulen | 152.26 | 2.74 | 0 | 0 | 29.16 | 0.03 |
| SCP105 | cineole | 152.26 | 2.16 | 0 | 1 | 59.96 | 0.05 |
