# Supplementary figures and images for: Enhancing and Complementary Mechanisms of Synergistic Action of Acori Tatarinowii Rhizoma and Codonopsis Radix for Alzheimer's Disease Based on Systems Pharmacology
Source: Evid Based Complement Alternat Med. 2020 Jun 25;2020:6317230. doi: 10.1155/2020/6317230 (PMC7334796; doi:10.1155/2020/6317230)

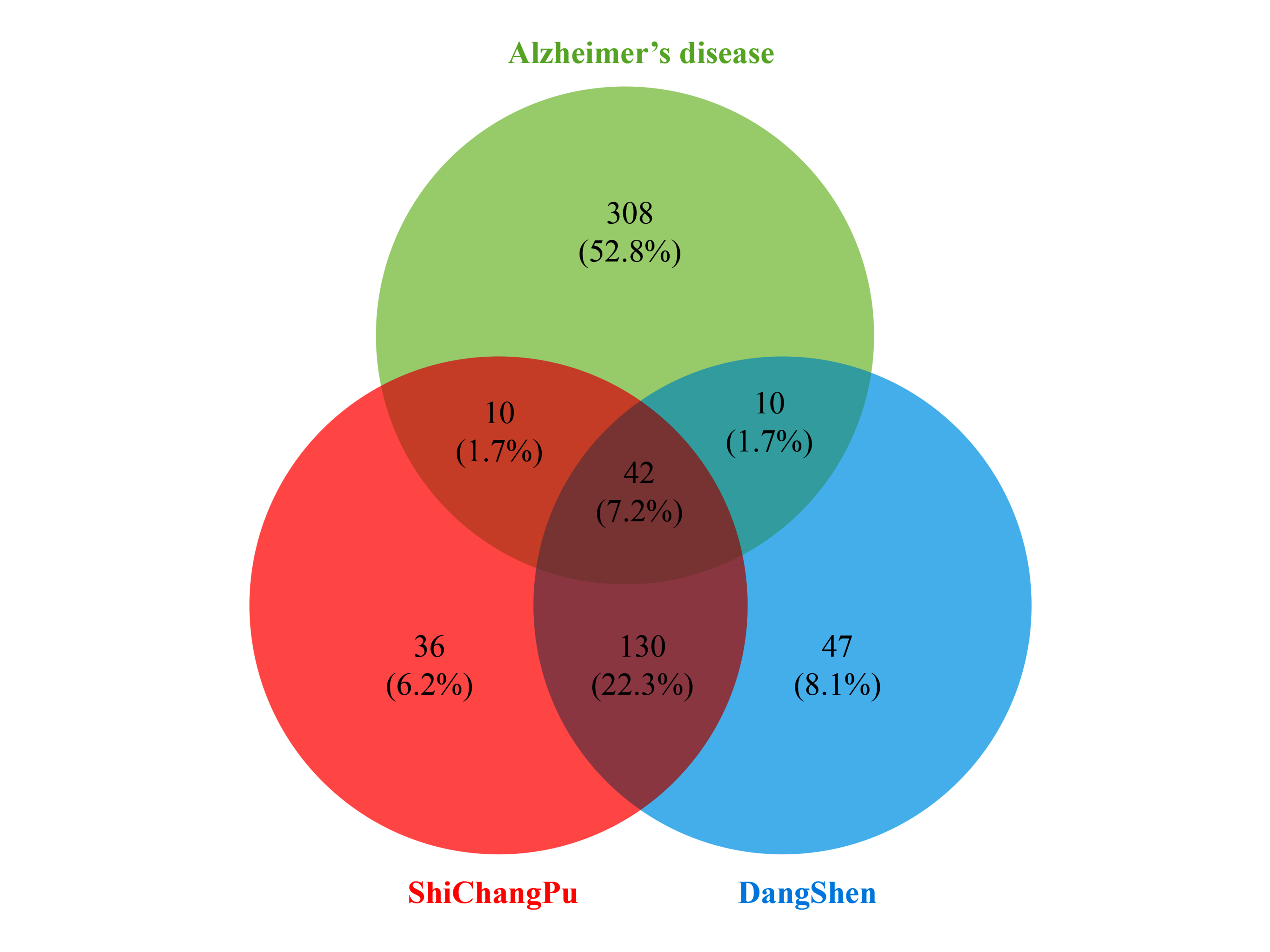

Supplement: Supplementary Materials — Figure S1. The number of targets of ATR (red), CR (blue), and Alzheimer's disease (green) obtained from databases. Figure S2. CS and accumulative CS of the active ingredients in ATR or CR. Table S1. Detailed information on the ingredients in ATR and CR. Table S2. The value of active ingredients in ATR and CR. [file 6317230.f1.zip › 6317230.f1/S1 Fig 1.tif]

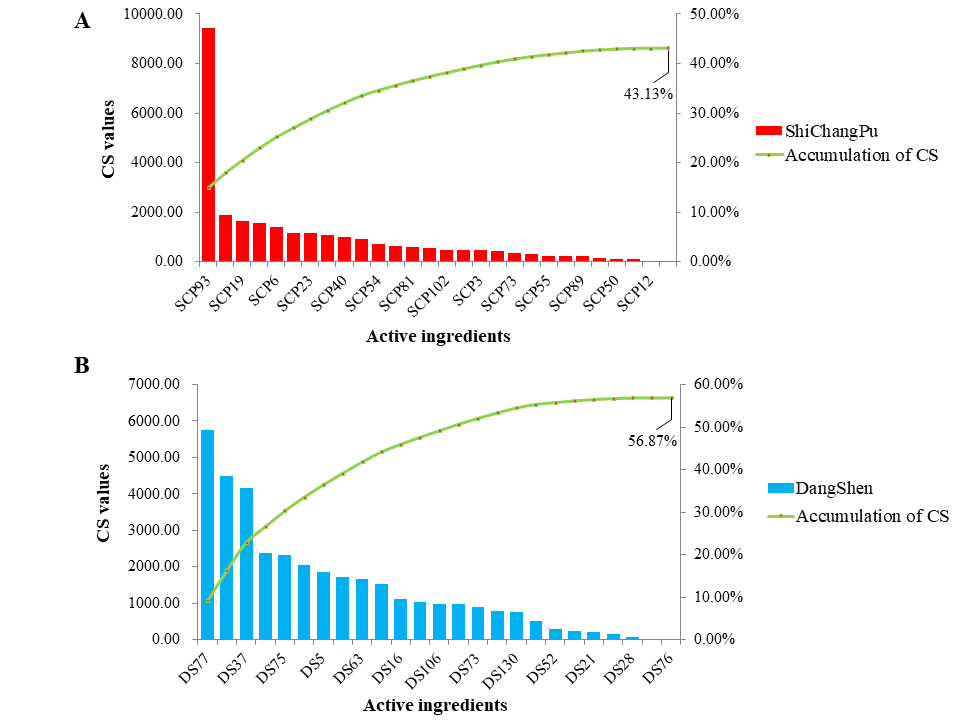

Supplement: Supplementary Materials — Figure S1. The number of targets of ATR (red), CR (blue), and Alzheimer's disease (green) obtained from databases. Figure S2. CS and accumulative CS of the active ingredients in ATR or CR. Table S1. Detailed information on the ingredients in ATR and CR. Table S2. The value of active ingredients in ATR and CR. [file 6317230.f1.zip › 6317230.f1/S2 Fig.tif]
